# Interactions Between High-Intensity Light and Unrestricted Vision in the Drive for Hyperopia

**DOI:** 10.1167/iovs.65.14.22

**Published:** 2024-12-10

**Authors:** Sayantan Biswas, Joanna Marie Fianza Busoy, Veluchamy A. Barathi, Arumugam R. Muralidharan, Leopold Schmetterer, Biten K. Kathrani, Noel A. Brennan, Raymond P. Najjar

**Affiliations:** 1 Singapore Eye Research Institute, Singapore, Singapore; 2 School of Optometry, College of Health and Life Sciences, Aston University, Birmingham, United Kingdom; 3 Ophthalmology and Visual Science Academic Clinical Program, Duke-NUS Medical School, Singapore, Singapore; 4 Yong Loo Lin School of Medicine, National University of Singapore, Singapore, Singapore; 5 Nanyang Technological University, Singapore, Singapore; 6 Johnson and Johnson, Jacksonville, Florida, United States; 7 Eye N' Brain Research Group, Department of Ophthalmology, Yong Loo Lin School of Medicine, National University of Singapore, Singapore, Singapore; 8 Department of Biomedical Engineering, College of Design and Engineering, National University of Singapore, Singapore, Singapore

**Keywords:** hyperopia, myopia, animal model, defocus, light, axial length, choroid

## Abstract

**Purpose:**

To evaluate the impact of optical vs. illuminance factors and their duration-dependency on lens-induced hyperopia (LIH) in chick eyes.

**Methods:**

Hyperopia was induced in one eye in chicks (10 groups; *n* = 126) from day 1 after hatching until day 8 using +10-diopter lenses with fellow eyes as controls. One group (LIH) served as the control without any interventions. The remaining groups were exposed to 2, 4, or 6 hours of unrestricted vision (UnV), high-intensity light (HL; 15,000 lux), or both (HL + UnV). Ocular axial length (AL), refractive error, and choroidal thickness were measured on days 1, 4, and 8. Inter-ocular difference (IOD = experimental − contralateral control eye) ± SEM was used to express outcome measures.

**Results:**

By day 8, LIH decreased AL (−0.42 ± 0.03 mm) and produced hyperopic refraction (+3.48 ± 0.32 diopters) and choroidal thickening (+85.81 ± 35.23 µm) in the LIH group (all *P* < 0.001). Exposure to UnV reduced LIH (i.e., hyperopic refraction, axial shortening, and choroidal thickening) in a duration-dependent manner, whereas HL potentiated the development of LIH in a duration-dependent manner. When combined, UnV overpowered HL, with resultant impact on refraction and AL being close to UnV alone, except at 6 hours, when HL + UnV induced shorter AL compared with UnV alone (*P* = 0.03).

**Conclusions:**

Daily exposure to HL, UnV, and HL + UnV altered LIH in a duration-dependent manner with UnV and LIH producing competing signals. The signal generated by UnV was generally stronger than HL in combined exposure, yet longer durations of HL affected the drive for emmetropization in eyes with UnV.

Emmetropization is a visually guided phenomenon, aiming to optimally focus the image on the retina throughout the development of the eye.^1^ Experimental myopic or hyperopic defocus using positive or negative lenses in front of the eye respectively, degrades the quality of the retinal image, disrupts normal emmetropization, leads to abnormal axial length (AL) of the eye,^3^^,^^4^ and leads to the development of refractive error.^2^^,^^3^

The most common refractive error is myopia or near sightedness. Myopia is a global epidemic with an exponential growth in its prevalence among children, adolescents, and young adults, especially in South and East Asia.^4^ In 2020, myopia affected nearly 30% of the world's population and this burden is expected to increase to 50% by 2050.^5^ Poor vision associated with myopia poses a global public health issue because it not only impacts the quality of early life, but also imposes socioeconomic consequences and increases the risk of sight-threatening conditions if left uncontrolled.^5^

Hyperopia is another type of refractive error characterized by hyperopic refraction and shorter AL of the eye.^6^ Humans are born hyperopic, and this condition remains relatively stable for hyperopic children throughout visual maturation.^7^ Both myopia and hyperopia can be induced in experimental animal models using negative or positive defocusing lenses, respectively.^8^ The lenses degrade the quality of the retinal image, and lead to abnormal AL of the eye^3^^,^^4^ and the development of refractive error.^2^^,^^3^ Myopic defocusing lenses (i.e., positive powered lens) fitted in front of the eye in animal models results in lens-induced hyperopia (LIH) associated with decreased ocular elongation, hyperopic refraction, and thicker choroid.^9^ Besides inducing hyperopia as a condition, positive lenses convey a “SLOW” growth signal to the eye.^10^ Study of this phenomenon may thus be useful in understanding and developing methods for controlling ocular growth, which may have application in the maintenance of hyperopic reserve, myopia prevention or slowing of myopic progression.^11^

Compared with the extensive research focusing on myopia development and progression, only a few studies have examined the development of hyperopia. In children, a transient thickening of the choroid is observed following 2 hours of myopic defocus (+3 diopters [D]),^12^ whereas a transient reduction in AL and associated choroidal thickening were observed in young adults with +3 D defocus within 15 to 60 minutes.^13^^,^^14^ Hence, incorporation of lens-induced myopic defocus as an optical correction can potentially control ocular growth and retard myopia progression in children. Results of long-term myopic defocus in the form of undercorrection of myopia, bifocals, and progressive addition spectacles are not clinically promising. Nonetheless, contact lenses with plus power in the lens periphery, orthokeratology—which induces peripheral plus corneal power—and spectacles with positively powered lenslets all have been shown to slow myopic progression.^15^

In addition to the optical “SLOW” growth signal, there is a growing body of evidence showing a protective effect of increased light intensity on the development of myopia, axial elongation and choroidal thinning in animal^16^^–^^20^ and clinical studies alike.^21^^,^^22^ Ashby et al.^17^ assessed the influence of high-intensity light (HL) on +7 D LIH on young chicks. They found HL to accelerate positive lens compensation (HL, 15,000 lux: +5.3 D vs. control 500 lux: +3.3 D) by day 3; however, by the end of the experiment (day 5), both the HL and control groups showed similar refraction in the eye subjected to LIH. Using dual powered lenses (+10 D/−10 D), Zheng et al.^23^ showed myopic defocus and HL to be additive against the myopiagenic hyperopic defocus.

Recently, we investigated the interactions between optical re-focus and HL in a lens-induced myopia model.^18^ Our findings suggest that HL (15,000 lux) and unrestricted vision (UnV) have a synergistic effect for reducing the development of lens-induced myopia (LIM) in chickens, only when administered for 6 hours. UnV of 2 to 6 hours were reported to effectively reduce 37% to 96% of LIM caused by hyperopic defocus.^18^^,^^24^ In contrast, based on Schmid and Wildsoet (1996), UnV was less potent in preventing LIH caused by myopic defocus, with only a 9% decrease after 3 hours of UnV in chickens.^24^ Equally, 3 hours of myopic defocus with 9 hours of UnV resulted in significant hyperopic refraction.^24^ Even wearing a positive lens for a brief period of 12 minutes per day and UnV otherwise developed hyperopia and reduced ocular elongation in chickens.^25^ In summary, UnV is less effective in slowing LIH than LIM in animal models.^26^ Similarly, findings from clinical studies also suggest myopic defocus to be more enduring than hyperopic defocus, producing stronger compensatory signals and greater persistence of the effects of myopic defocus even after its cessation.^27^

To date, the duration-dependent and synergistic effect of HL and UnV is yet to be studied in an LIH animal model. In this study we explore the duration-dependent effect of (1) myopic defocus, (2) HL, (3) UnV, and (4) their combinations on hyperopia development (i.e., the SLOW signal for ocular growth).

## Methods

### Animals and Experimental Setup

The animals used in this study were treated in accordance with the ARVO statement for the Use of Animals in Ophthalmic and Vision Research. The study protocol (IACUC 2019/SHS/1479) was approved by the Association for Assessment and Accreditation of Laboratory Animal Care International accredited Singapore Experimental Medicine Centre Institutional Animal Care and Use Committee.

A total of 126, 1-day-old chicks (mixed Golden Comet/White Leghorn strain) were obtained from the National Large Animal Research facility and were randomly divided into 10 groups, with each group consisting of 11 to 13 animals. The chicks were raised for 9 days in a custom-built enclosure of 75 cm (length) × 55 cm (width) × 43 cm (height) designed to hold two high-intensity light-emitting diode (LED) light fixtures. A light-dark cycle of 12/12 hours was kept from 7 AM to 7 PM, and the temperature maintained between 28°C and 32°C within the enclosure with food and water provided ad libitum. A HOBO Pendant data logger (UA-022-64; ONSET, Bourne, MA, USA) was used to monitor the light and temperature patterns. Square wave gratings of a repeated sequence of light and dark bars were fitted on the enclosure wall as accommodative cues. Depending on the location of the animal within the enclosure, the spatial frequency of the gratings ranged between 0.01 to 0.42 cycles/degree. To ensure that emmetropization in chicks is not affected by variations in accommodative responses,^28^ all experimental groups were exposed to an identical visual environment. On the final day 9 of the experiment, the chicks were administered a sedative mixture of 0.2 mL/kg ketamine and 0.1 mL/kg xylazine. Subsequently, they were euthanized by administering an overdose of sodium pentobarbitone directly to the heart.

### Background and Experimental Light Setup

Throughout the 12/12-hour light–dark cycle, all chicks were raised under background lighting conditions of 150 lux. Light parameters were measured in all directions of gaze (i.e., chicken looking up, 399.6 lux; right, 115.7 lux; left: 114.5 lux; front, 97.1 lux; rear, 98.9 lux and down, 102.7 lux) and averaged. To achieve this, six strips of ultra-bright LEDs (4000K, 2NFLS-NW LED; Super Bright LED, Inc, St. Louis, MO, USA) were securely positioned above the enclosure. For the HL group, 4 LED panels, each consisting of 64 LEDs, were used, providing an average of 15,000 lux when measured at chicken eye level for various gaze angles (up, right, left, front, rear, and down) within the enclosure. The lighting system was controlled by a programmable Helvar DIGIDIM 910 router (Helvar, Dartford Kent, UK). To ensure accuracy, light levels and spectra were assessed using a calibrated radiometer and spectroradiometer (ILT5000 and ILT950; International Light Technologies, Peabody, MA, USA).

### Hyperopia Induction

Hyperopia was induced monocularly in all chicks from day 1 after hatching until day 8. This was achieved by utilizing a customized convex defocusing lens (La SER Eye Jewelry, Port St. Lucie, FL, USA) with a power of +10 ± 0.5 D. The lenses had a total diameter of 12.5 mm and an optic zone diameter of 10 mm, with a base curve of 6.68 mm. The lens-treated eye was selected randomly for each animal and fitted with a three-dimensional printed lens holder, custom designed for this purpose. To secure the positioning of the lenses on the chick's eyes and facilitate removal during cleaning and light exposure (in some groups), the lens holders were attached to a separate base piece that was glued down to the feathers surrounding the eye. Taking into consideration the 10-mm diameter of the optic zone, an estimated vertex distance of 3 mm (from the defocusing lens to the corneal apex), and a calculated distance of 4.49 mm from the posterior nodal point to the defocusing lens on day 1 in chicks, the approximate open viewing visual angle was estimated to be approximately 76.5° degrees. However, it should be noted that the open viewing visual angle might have been underestimated, because these calculations did not account for changes in pupil size.^29^ The lenses were worn for a total of 8 days and were thoroughly cleaned three times per day to maintain their optical clarity.^8^^,^^30^ The fellow eye remained uncovered and served as a control within each individual animal.

### Experimental Groups

Monocular LIH was applied to all the 10 groups of chicks. Out of these, nine groups underwent various interventions, such as HL (15,000 Lux), UnV, or a combination of HL and UnV, each lasting for different durations (0, 2, 4, or 6 hours) centered at 12:00 pm. Further information regarding the experimental interventions can be found elsewhere in this text and in Table.

**Table.** Details on Experimental Groups and Interventions

					Experimental Interventions		
Experimental Group	Duration of Intervention (Hours)	No.	Experimental Eye	Control Eye	High-intensity Light Status (15,000 lux)	Lens Status	Time of Intervention
LIH	0	13	+10 D	No lens	Off	Not removed	-
LIH + HL	2	13	+10 D	No lens	On	Not removed	11:00–13:00
	4	13	+10 D	No lens	On	Not removed	10:00–14:00
	6	13	+10 D	No lens	On	Not removed	09:00–15:00
LIH + UnV	2	13	+10 D	No lens	Off	Removed	11:00–13:00
	4	13	+10 D	No lens	Off	Removed	10:00–14:00
	6	12	+10 D	No lens	Off	Removed	09:00–15:00
LIH + HL + UnV	2	11	+10 D	No lens	On	Removed	11:00–13:00
	4	12	+10 D	No lens	On	Removed	10:00–14:00
	6	13	+10 D	No lens	On	Removed	09:00–15:00

### LIH Group

A total of 13 chicks in this group were raised in background laboratory light conditions (150 lux), and they were not exposed to HL or UnV.

### High-Intensity Light Groups (LIH + HL)

All 3 groups had 13 chicks each and were exposed to 2, 4, or 6 hours of HL (15,000 lux) every day without removal of the defocusing lenses and background light for the remainder of the light cycle.

### Unrestricted Vision Groups (LIH + UnV)

Defocusing lenses were removed for 2, 4, or 6 hours per day for the 3 groups (*n* = 13, 13, and 12). These groups were raised under background lighting conditions throughout the experiment.

### High-Intensity Light and Unrestricted Vision Groups (LIH + HL + UnV)

The 3 groups (*n* = 11, 12, and 13) were exposed to 2, 4, or 6 hours of HL (15,000 lux) every day without removal of the defocusing lenses. The groups were exposed to background light for the remainder of the light cycle.

### Ocular Measurements In Vivo

All ocular measurements were carried out in a dimly lit room (<5 lux) between 12 pm and 5 pm and the animals were evaluated randomly to reduce the impact of circadian rhythm on the outcome measures. The body weight, ocular AL, refractive error, choroidal thickness (CT), central corneal thickness (CCT), and anterior chamber depth (ACD) were measured in all animals on day 1, day 4, and day 8 after the protocol, which is described elsewhere.^18^^,^^31^ A few chicks (2 animals on day 1) who could not keep their eyelids open needed a lid retractor. The examiner carefully instilled a drop of topical anesthetic (proparacaine [Alcaine] Alcon Laboratories, Inc. Fort Worth, TX, USA) and inserted the lid retractor without touching the cornea or obstructing the examination procedure. This procedure was carried out in alert chicks by handling them gently. Anesthesia was avoided during all ocular measurements to prevent potential fatality in 1-day-old animals and to avoid potential ocular changes that may occur with anesthesia.^32^

### Axial Length

VuMAX HD (Sonomed Escalon, New Hyde Park, NY, USA) A-scan ultrasound was used to measure the AL as described by Najjar et al.^31^ In summary, the AL was defined as the distance between the echo spike originating from the anterior surface of the cornea and most anterior spike originating from the retina at a probe frequency of 10 MHz. A median of 7 to 10 scans were recorded as an individual reading.

### Refraction

An automated infrared photoretinoscope (calibrated for a range of −10 D to +10 D) was used as previously described^33^ to measure ocular refraction. The chicks were gently held on an adjustable platform placed approximately 1 m away from the infrared photorefractor. The positioning of the chick's head was done with great care to ensure optimal focus on its eye and to detect the first Purkinje image. The automated infrared photoretinoscope measures continuous refraction traces. The median of the most hyperopic refraction readings (i.e., resting refraction) with no accommodative changes (stable refraction) were isolated by visualizing the graphs, as recommended by the instruction manual of the photoretinoscope (by Professor Frank Schaeffel) and performed in our previous publications.^18^^,^^34^ Each time point comprising of at least 300 readings in each eye.

### Choroidal Thickness and Anterior Segment

Posterior segment spectral-domain optical coherence tomography (OCT; Spectralis; Heidelberg Engineering, Inc., Heidelberg, Germany) was used to measure the CT, whereas anterior segment OCT (RTVue; Optovue, Inc., Fremont, CA, USA) was used to image the anterior segment (ACD and CCT) as per the protocols described in Najjar et al.^31^ For posterior segment OCT measurements, the procedure was performed as indicated by Lan et al. (2013)^35^ and Najjar et al. (2021).^31^ In brief, the chicken was handled gently, and their eye was aligned with the optics of the OCT to let the infrared laser beam to enter the eye through the center of the pupil. The centration and alignment of the pupil was refined before obtaining multiple OCT scans.. The axial and lateral resolutions of the system were 3.87 µm and 5.42 µm, respectively. Tracking was not used because retinal structures are not visible in young chicks on OCT. Once proper alignment and fixation of the pupil was confirmed (see Supplementary Fig. S1) by the operator, multiple OCT single scans were captured for each eye at the posterior poles (30°). Subsequently, only the scan with highest quality and in which the images were properly centered (within ±100 µm from the horizontal line) and clearly visible were used for further analysis. Three measurements of the CT were taken: one at the central position of the scan and two more at 1.0 to 1.5 mm around the center. CT measurements (*n* = 3 per image) were averaged from at least two eligible scans per eye. The CT was defined as the distance between the inner border of the sclera and the outer border of the retinal pigment epithelium. The distance between the central most posterior layer of the cornea and the central most anterior layer of the lens was defined as the ACD, whereas the CCT was defined as the average of three thickness measurements of the central cornea. The first author (S.B.), who was kept masked to the condition of treatment throughout the measurement sessions, performed all the measurements manually.

### Analyses and Statistics

The data are presented as the mean ± SEM of the interocular difference (IOD) between the experimental (LIH) and the control eye (uncovered) and calculated as the LIH eye – control eye. This approach accounts for the interanimal variations in outcome measures due to the mixed breed and large number of animals (*n* = 126 chicks) included in this study. For comparing IODs in refraction, AL, CT, ACD, and CCT, a two-way repeated-measures ANOVA was used. The factors considered were day, group, and the interaction between group and day. In case where the omnibus test indicated a significant interaction effect between group and day, pairwise multiple comparisons were conducted using the Holm–Sidak method. A two-way ANOVA was performed to assess the interaction between the type of intervention (HL, UnV, HL + UnV) and its duration (0, 2, 4, and 6 hours) on the refraction, AL, and CT. If the omnibus test yielded statistical significance, pairwise multiple comparisons were conducted using the Holm–Sidak method. For all statistical tests, the significance level was set at α = 0.05, and Sidak correction was applied for post hoc pairwise comparisons.

## Results

### Ocular Changes Associated With LIH

**Figure 1.**
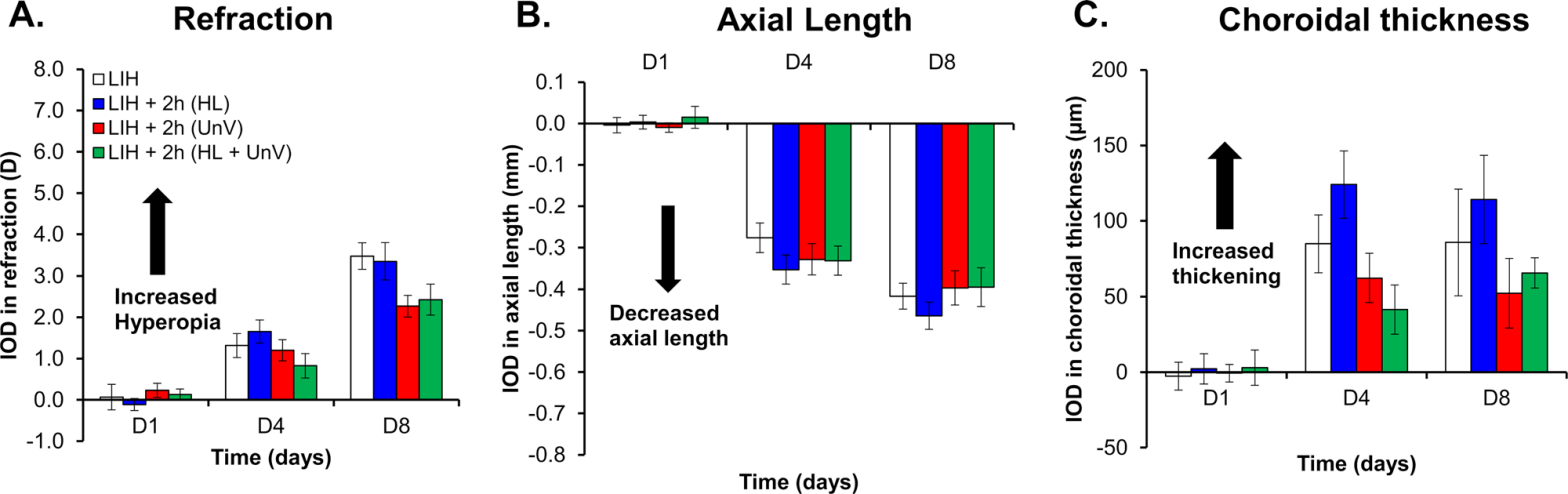
IOD in refraction, AL, and CT on days 1, 4, and 8 of the experimental protocol in the group not exposed to any intervention (LIH) and groups exposed to 2 hours of HL, UnV, or both (HL + UnV).

**Figure 2.**
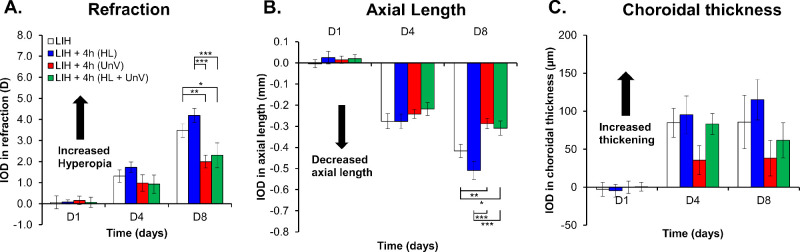
IOD in refraction, AL, and CT on days 1, 4, and 8 of the experimental protocol in the group not exposed to any intervention (LIH) and groups exposed to 4 hours of HL, UnV, or both (HL + UnV). For significant group effect: *P < 0.05, **P < 0.01, ***P < 0.001 (two-way repeated-measures ANOVA).

**Figure 3.**
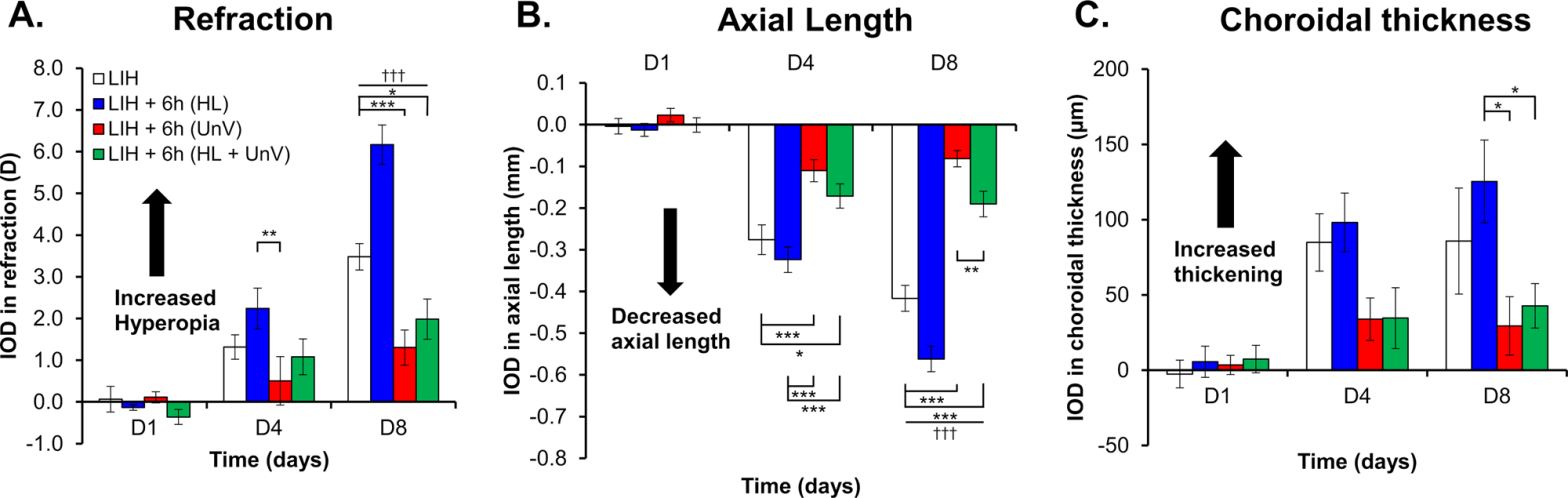
IOD in refraction, AL, and CT on days 1, 4, and 8 of the experimental protocol in the group not exposed to any intervention (LIH) and groups exposed to 6 hours of HL, UnV, or both (HL + UnV). All groups are significantly different from the LIH + HL group: ^†††^*P <* 0.001. For significant group effect: *P < 0.05, **P < 0.01, ***P < 0.001 (two-way repeated-measures ANOVA).

The LIH eyes developed hyperopic shift in refractive error (refraction, +5.12 ± 0.24 D and +7.39 ± 0.36 D by day 4 and day 8, respectively), primarily within the initial 4 days of +10 D lens wear (IOD, +1.31 ± 0.29 D and +3.48 ± 0.32 D by day 4 and day 8, respectively), in comparison with the uncovered contralateral control eyes (refraction, +3.81 ± 0.29 D and +3.91 ± 0.13 D by day 4 and day 8, respectively). Simultaneously, there was a reduced axial elongation in the LIH eyes (IOD, −0.28 ± 0.04 mm and −0.42 ± 0.03 mm by day 4 and day 8, respectively) and an increase in CT (IOD, +84.85 ± 19.05 µm and +85.81 ± 35.23 µm by day 4 and day 8, respectively) compared with the control eyes (all *P* < 0.001) (Figs. 1, 2 and 3, and Supplementary Table S1). Please note that LIH histograms are the same across Figures 1 through 3. There was no difference in the CCT and ACD between LIH and control eyes (Supplementary Figs. S2 and S3).

### Impact of 2 Hours of HL, UnV, and HL + UnV

For the 2-hour interventions, IOD in refraction, F(2,46) = 82.53, *P* < 0.001 (Fig. 1A); AL, F(2,46) = 221.31, *P* < 0.001 (Fig. 1B); and CT, F(2,46) = 25.67, *P* < 0.001 (Fig. 1C) were only significantly different between the days of the intervention. Detailed results are available in Supplementary Table S1.

### Impact of 4 Hours of HL, UnV, and HL + UnV

Four-hour interventions showed significant interactions between experimental group and day for IOD in refraction, F(6,92) = 2.38, *P* = 0.035 (Fig. 2A). By day 8, both 4 hours of UnV and HL+ UnV significantly reduced hyperopic refraction compared with the LIH group (both *P* < 0.05). UnV and HL+ UnV were equally effective (P > 0.05) in reducing hyperopia. HL, in contrast, significantly increased hyperopic refraction compared with both UnV and HL + UnV (both *P* < 0.001) (Fig. 2A). The group × day interaction was significant also for IOD in AL, F(6,94) = 4.59, *P* < 0.001. Similar to reducing hyperopic refraction, by day 8, 4 hours of UnV and HL+ UnV significantly increased AL elongation compared with the LIH only group (both *P* < 0.05). Equally, HL was significantly effective in reducing axial elongation compared with both UnV and HL + UnV (both *P* < 0.001) (Fig. 2B). IOD in CT was only dependent on the day of the intervention, F(2,94) = 21.34, *P* < 0.001 (Fig. 2C). Detailed results are available in Supplementary Table S1.

### Impact of 6 Hours of HL, UnV, and HL + UnV

For the 6-hour interventions, there was a significant Group × Day interaction for IOD in refraction, F(6,94) = 9.64, *P* < 0.001. By day 8, 6 hours of UnV (*P* < 0.001) and HL + UnV (*P* = 0.011) significantly reduced hyperopic refraction, whereas HL alone increased hyperopic refraction compared with the LIH group (*P* < 0.001). HL significantly increased hyperopic refraction compared with UnV on day 4 and day 8 (both *P* < 0.01) and compared with HL + UnV on day 8 (*P* < 0.001) (Fig. 3A). IOD in AL showed a significant group × day interaction, F(6,94) = 17.40, *P* < 0.001, with UnV and HL + UnV showing increased axial elongation compared with the LIH eyes on day 4 and day 8 (LIH vs. UnV: *P* < 0.001 and LIH vs. HL + UnV: *P* < 0.05). On day 8, HL produced significantly more reduction in AL compared with LIH (*P* < 0.001). On both day 4 and day 8, HL exposed eyes had greater AL reduction than both UnV and HL + UnV (all *P* < 0.001) (Fig. 3B). IOD in CT was dependent on the group, F(3,94) = 4.04, *P* = 0.012, and day, F(2,94) = 17.61, *P* < 0.001, individually, but their interactions did not reach statistical significance. CT in eyes exposed to HL was significantly higher than those with UnV (*P* = 0.024) or HL + UnV (*P* = 0.042) (Fig. 3C). Detailed results are available in Supplementary Table S1.

### Impact of Experimental Interventions on ACD and CCT

IODs in ACD showed a significant effect of day for the 2-hour, F(2,92) = 21.75, *P* < 0.001, 4-hour, F(2,94) = 13.99, *P* < 0.001, and 6-hour, F(2,94) = 20.34, *P* < 0.001, interventions. IODs in CCT showed a significant effect of day only for 4-hour, F(2,94) = 4.12, *P* = 0.019, and 6-hour, F(2,94) = 7.39, *P* = 0.001, interventions (Supplementary Figs. 2 and 3). For detailed results, see Supplementary Table S1.

### Duration Response Curves on day 4 and day 8 of the Interventions

On day 4, the impact of intervention on IODs in refraction, F(2,104) = 6.02, *P* = 0.003, was not duration dependent. For refraction, groups exposed to HL had significantly higher hyperopic refraction compared with those with UnV and HL + UnV (HL vs. UnV: *P* = 0.008, HL vs. HL + UnV: *P* = 0.009) (Supplementary Fig. 4A). The impact of the intervention on IODs of AL, F(2,104) = 14.15, *P* < 0.001, was duration dependent. The interaction between the group and duration for IOD in AL was significant, F(4,104) = 2.98, *P* = 0.023, where 6 hours of HL was more effective in reducing ocular elongation than UnV (*P* < 0.001) and HL + UnV (*P* = 0.001) (Supplementary Fig. 4B). IODs in CT were different between the intervention groups, F(2,104) = 9.36, *P* < 0.001, with eyes exposed to HL having significantly thicker choroids than eyes exposed to UnV and HL + UnV (HL vs. UnV: *P* < 0.001, HL vs. HL + UnV: *P* = 0.002) (Supplementary Fig. 4C).

**Figure 4.**
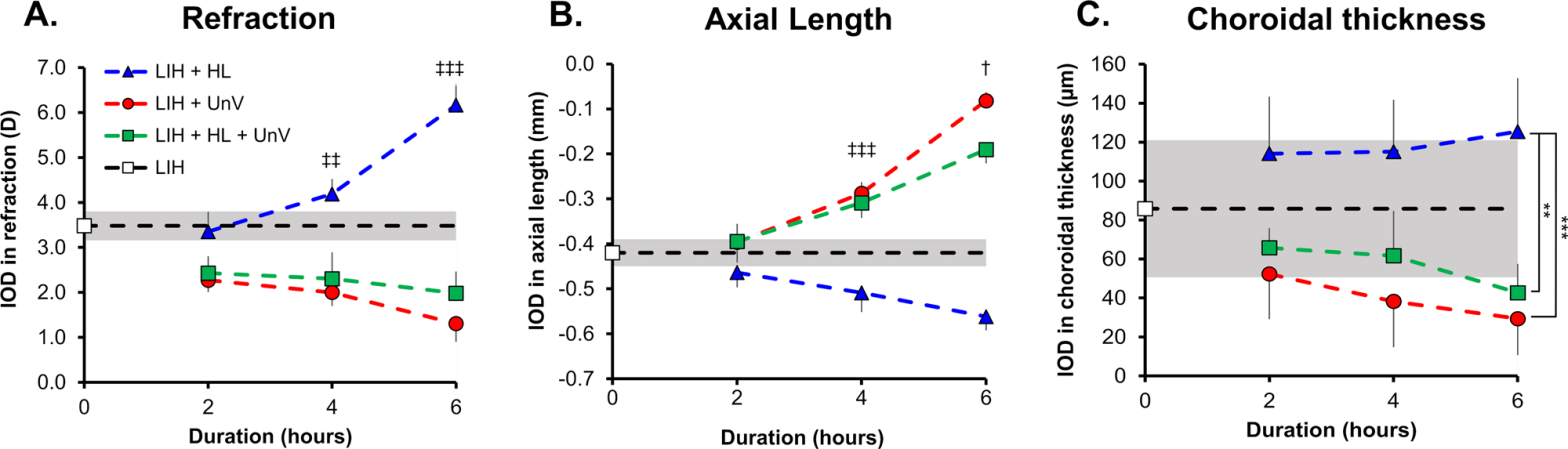
Duration–response curve for the IOD in refraction (**A**), AL (**B**), and CT (**C**) in the groups exposed to 2, 4, and 6 hours of HL, UnV, or both (HL + UnV) on day 8 of the experimental protocol. The LIH group that was not exposed to any intervention is represented by a *white square* and a *shaded area* for mean ± 95% confidence interval. HL is significantly different from the other two groups: ^‡‡^*P <* 0.01, ^‡‡‡^*P* < 0.001. All the groups are different from each other: ^†^*P <* 0.05 (at least). The HL group is significantly different from both UnV and HL + UnV groups: *P < 0.05, **P < 0.01, ***P < 0.001.

On day 8 of the protocol, there was a significant interaction between the duration and type of intervention on IODs of refraction, F(4,104) = 7.07, *P* < 0.001. Both 4 hours and 6 hours of HL, but not 2 hours of HL, significantly increased hyperopic refraction induced by LIH compared with UnV (both 4 and 6 hours: *P* < 0.001) and HL + UnV (4 hours, *P* = 0.003 and 6 hours, *P* < 0.001), which decreased hyperopic refraction compared with LIH (4 hours, both UnV and HL + UnV: *P* < 0.05; 6 hours, UnV: *P* < 0.001 and HL + UnV: *P* = 0.011) (Fig. 4A). Likewise, the interaction between the duration and type of intervention was significant for AL, F(4,104) = 9.87, *P* < 0.001, where both 4 hours and 6 hours of HL, but not 2 hours of HL, further reduced AL compared with UnV (both 4 and 6 hours, *P* < 0.001) and HL + UnV (both 4 and 6 hours, *P* < 0.001), which increased the AL compared with the LIH group (prevented AL shortening) (4 hours, both UnV and HL + UnV: *P* < 0.05; 6 hours, both UnV and HL + UnV: *P* < 0.001). For the 6-hour group, experimental eyes exposed to HL +UnV had shorter AL compared with eyes exposed to UnV (*P* = 0.028) (Fig. 4B). IODs in CT, F(2,104) = 9.75, *P* < 0.001, was different between groups across the different durations of the interventions, with HL inducing further choroidal thickening compared with LIH and compared with UnV (*P* < 0.001) and HL + UnV (*P* = 0.003) (Fig. 4C).

## Discussion

In this study, we investigated the duration-dependent, differential, and combined effects of HL and UnV on the ocular growth SLOW signal induced by LIH in a chicken model. The effect of HL, UnV, and HL + UnV in altering hyperopic refraction, AL elongation, and CT were duration dependent by day 8 of the intervention. Unlike in LIM, HL and UnV did not yield a similar effect in an LIH model. As previously reported,^18^ HL exacerbated the effects of LIH (i.e., increased hyperopic refraction, axial shortening and choroidal thickening) in a duration-dependent manner, with the greatest impact observed after 6 hours of exposure, followed by 4 and 2 hours. Conversely, UnV countered the effects of LIH (i.e., reduced hyperopic refraction, axial shortening, and choroidal thickening) in a duration-dependent manner with the greatest being after 6 hours of exposure, followed by 4 and 2 hours. Interestingly, the impact of UnV was greater than that of HL. The combined effects of HL + UnV were similar to those of UnV alone. However, after 6 hours of exposure to HL + UnV, the eyes exposed to LIH + HL + UnV had shorter ALs compared with those exposed to LIH + UnV. Consistent with previous findings, there was no significant change in ACD or CCT among the groups.^17^ See Supplementary Fig. S2 for a detailed description of the literature investigating the effect of HL and UnV on LIH.

The effect of UnV in reducing LIH in a duration-dependent manner has been reported previously by Schmid et al.,^24^ where hyperopic refraction decreased by 8.4%, 27.7%, and 42.2% on exposure to 3, 6, and 9 hours of UnV by D5, respectively. Correspondingly, exposure to UnV for 3, 6, and 9 hours per day increased AL elongation by 11.1%, 22.2%, and 44.4%, respectively.^24^ In comparison, by day 8 we observed a 34.8%, 42.5%, and 62.6% decrease in hyperopic refraction and a 4.8%, 31.0%, and 81.0% increase in AL elongation on exposure to 2, 4, and 6 hours of UnV, respectively. The increased impact of UnV observed in our study potentially could be attributed to disparities in the experimental protocol, such as the age (visual maturation), strain of chickens, and duration of the experimental protocol, as well as background lighting, visuospatial surroundings during UnV and the timing of UnV (centered around noon for this study and spilling into the afternoon). In fact, 2 hours of myopic defocus (+10 D) applied at noon or in the evening is more effective at reducing ocular growth compared with continuous or morning-only myopic defocus.^36^ Similarly, 2 hours of positive lens removal at noon and in the evening caused increase in ocular growth more than morning removal.^36^ When it comes to the temporal dynamics of hyperopia induction, it has been proposed that temporal changes induced by compensation to positive lenses, although duration dependent, is nonlinear, because the rise and fall of the internal emmetropization signal is not directly proportional to the duration of lens wear, but rather based on the frequency of wear with short durations.^26^ In addition, earlier studies investigating the impact of UnV on LIH reported that interrupted hyperopia (UnV = 2 hours of relief from +4 D) resulted in a myopic shift in refractive state compared with the constant hyperopic group in tree shrews.^37^ These findings, along with ours, suggest that UnV pushes toward emmetropization based on the updated (i.e., the temporary hyperopic defocus created during UnV) state of image defocus. Conversely, using +5 D lens wear, Zhu et al.^38^ showed that even 30 minutes of UnV twice a day can result in a 43% increase in hyperopia in marmosets. These findings, although contradictory to ours, suggest that the inherent emmetropization signal to low myopic defocus (+5 D) does not decay when the treatment period is long (4 weeks) accompanied by multiple visual stimulation (UnV/ LIH × twice a day).

Exposing LIH eyes (+7 D) to HL (15,000 lux) for 5 hours per day, Ashby and Schaeffel^17^ showed a significant change in refraction by day 3 and day 4 accompanied by reduction in AL elongation compared with LIH eyes without HL. However, by day 5 there was no change in refraction, but a 46.2% reduction in AL elongation was recorded by day 6. In contrast, we recorded a −3.7%, 20.4%, and 77.3% increase in hyperopic refraction and a 9.5%, 21.4%, and 33.3% reduction in AL elongation relative to the contralateral control eye by day 8 on exposure to 2, 4, and 6 hours of HL, respectively. We expected a significant difference between LIH and LIH + HL on day 4, as reported by Ashby and Schaeffel (2010).^17^ Although we did observe a trend toward more hyperopic refraction and a shorter AL in eyes exposed to HL on day 4 of the experiment, this trend was not significant. This result may be attributed to the differences in experimental protocol, ambient illumination levels, and the type of experimental lights used in the studies. Ashby and Schaeffel^17^ used light that mimicked daylight (range, 300–1000 nm; peak, 700 nm), whereas our experimental lights had typical LED spectrum with two peaks at approximately 449 nm and 583 nm. Recently, a study on form deprivation myopia has shown that the fullness of light spectrum may affect the refractive development in chicks.^34^ Light measurement in animal studies currently lacks standardization, with varied metrics and methods of measurement reported. Efforts are underway to standardize light reporting in animal models^39^ and humans.^40^ Future research should adopt these standardized methods to improve data comparability and enhance meta-analyses. In addition, other influencing factors might be spatial frequency of the enclosure (low spatial frequency vs unclear spatial frequency in Ashby and Schaeffel^17^), chick age (1 day vs 7 days in Ashby and Schaeffel^17^), strain difference, sample size differences, or a potential threshold for the maturation of the chick retina. Even though the chick retina is considered mature from the day of hatching (after embryonic day 21),^41^^,^^42^ the visual system continues to mature until day 8 (after hatching).^43^

Nevertheless, our study agrees with Ashby et al.’s^17^ findings at day 4 on the concept that HL potentiates LIH and axial shortening, whereas UnV promotes emmetropization based on the updated ocular defocus status (i.e., the hyperopic eye without the positive lens), thus slowing LIH. Whether HL would still promote AL shortening had emmetropization been achieved (+10 D) is unclear. Yet, 6 hours of HL when combined with UnV triggered AL shortening compared with UnV alone (Fig. 3B), thus suggesting that HL always promotes AL shortening rather than ocular compensation to defocus. These findings may explain a role of HL outdoors in protecting against myopia, through a potential build-up and maintenance of a hyperopic reserve in growing eyes.

The choroid plays a role in the regulation of ocular growth and emmetropization. Choroidal thickening occurs in response to myopic defocus (positive lens).^44^^,^^45^ Although studies on the effect of HL on CT under LIH are lacking, HL without LIH is expected to induce an increase in CT.^18^^,^^34^^,^^35^ Yet, we did not observe any increase in the CT of control eyes exposed to HL (i.e., HL and HL + UnV) compared with control eyes not exposure to HL (i.e., UnV). Conversely, HL in addition to positive lens, led to significantly thicker choroid compared with HL + UnV and UnV. This change in CT is thought to be largely due to change in choroidal blood flow, permeability and vasodilation of choroidal vessels associated with the rise in intraocular temperature and neurotransmitter release.^46^^,^^47^ By day 8, LIH eyes exposed to 2, 4, and 6 hours of HL had choroidal thickening by 33%, 34.2%, and 46.2% respectively, whereas eyes exposed to 2, 4, and 6 hours of HL + UnV and UnV had choroidal thinning by 23.4%, 28.1%, and 50.3% and 39%, 55.5%, and 65.8%, respectively. Even though both HL + UnV and UnV resulted in decreased CT, HL + UnV had a slightly thicker choroid than UnV alone (*P* > 0.05) (Figs. 1^2^–3C). Contrary to our finding, choroidal thickening by 16% was observed on removal of the myopic defocus (+5 D) for 30 minutes twice a day in marmoset eyes.^38^ According to Zhu et al. (2022),^38^ interrupting positive lens wear during periods of normal vision might have altered how the eye's growth control system response, potentially leading to an intensified or inflated reaction to normal vision. Another possibility is that these interruptions amplified the visual system's gain or sensitivity, causing it to respond more rapidly to signals in between episodes of lens wear.

HL and UnV probably trigger different mechanisms of action. UnV, being a visual/optical feedback guided phenomenon,^9^^,^^48^ stops emmetropizing the eye at null IODs. In contrast, HL seems to work via a different pathway involving photoreceptor stimulation and releasing of retinal neurotransmitters.^16^^,^^17^^,^^49^ HL induced increase in retinal dopamine (DA) level is associated with lower LIM.^50^ However, the role of DA in positive lens compensation is unclear with mixed reports of both enhancement^51^ and no effect^52^ on LIH with injection of DA agonist such as apomorphine and 6-hydroxy DA, respectively. Studying the possible dopaminergic and cholinergic mechanisms of LIH development resulted in contradictory findings of increase,^53^ decrease or no change ^54^^,^^55^ in retinal DA levels in eyes with LIH. Gamma-aminobutyric acid is another neurotransmitter related to the light exposure and is coreleased alongside DA from the dopaminergic amacrine cells.^56^ Baclofen, a gamma-aminobutyric acid B receptor agonist administration reduces LIH and CT, which further inhibits DA release and 3,4-dihydroxy-phenylacetic acid content compared with LIH eyes without baclofen.^55^

Our study has a few limitations. First, it is difficult to generalize our findings in chicks to humans given the differences between chicken and humans in their ocular anatomy and optics.^57^ The chicks were housed in a visual environment devoid of fine spatial details, color, and other regular features, which promotes emmetropization.^58^ Although the findings are in alignment with the literature suggesting that removing myopic defocus reduces hyperopia development, the finding is limited to animal models because humans are not subjected to myopic defocus in daily life. The other finding is that exposure to HL can potentiate hyperopia development in a duration-dependent manner regardless of the optical status of the eye. However, exposure to such high intensity (15,000 lux) of light for 16%, 33%, or 50% (2, 4, or 6 hours) of the daytime is often not implementable in real life.

## Conclusions

Our study showed that daily exposure to 2, 4, or 6 hours of UnV slows LIH by promoting emmetropization in a duration-dependent manner. The combination of UnV and HL of 2 to 4 hours does not potentiate the impact of UnV. Conversely, our findings suggest that HL potentiates the drive for hyperopia (slowing ocular growth) independent of the optical status of the eye. From a translational perspective, our findings also indirectly highlight the capability of long periods of exposure to HL to secure a hyperopic reserve in developing eyes, which may explain the protective effect of time outdoors against myopia onset.

## Supplementary Material

Supplement 1
