# Supplementary material for: Interactions Between High-Intensity Light and Unrestricted Vision in the Drive for Hyperopia
Source: Invest Ophthalmol Vis Sci. 2024 Dec 10;65(14):22. doi: 10.1167/iovs.65.14.22 (PMC11645742; doi:10.1167/iovs.65.14.22)
Supplement: Supplement 1 [file iovs-65-14-22_s001.pdf]

Supplementary Material

Interactions between high-intensity light and unrestricted vision in the drive for hyperopia

Sayantan Biswas[1,2], Joanna Marie Fianza Busoy[1], Veluchamy A. Barathi[1,3,4], Arumugam R. Muralidharan[1,3], Leopold Schmetterer[1,5], Biten K. Kathrani[6], Noel A. Brennan[6], Raymond P. Najjar[1,3,7,8 *]

Supplementary Tables

Supplementary Table S1: Ocular measurements in groups exposed to 0h, 2h, 4h and 6h of high intensity light, unrestricted vision or both. Data represented as mean ± SEM of the interocular difference between experimental and control eyes.

Ocular parameter	Duration of intervention (hours)	Protocol	Experimental eye			Control eye			IOD (Experimental - Control)			*P-values 2W RM ANOVA		
			**D1**	**D4**	**D8**	**D1**	**D4**	**D8**	**D1**	**D4**	**D8**	**Group**	**Day**	**Group × Day**
Refraction (D)	0	LIH	4.72 ± 0.23	5.12 ± 0.24	7.39 ± 0.36	4.65 ± 0.23	3.81 ± 0.29	3.91 ± 0.13	0.07 ± 0.31	1.31 ± 0.29	3.48 ± 0.32	-		
Axial length (mm)			7.45 ± 0.06	7.45 ± 0.06	7.49 ± 0.06	7.45 ± 0.05	7.72 ± 0.04	7.90 ± 0.06	0.00 ± 0.02	-0.28 ± 0.04	-0.42 ± 0.03			
Choroidal thickness (µm)			167.50 ± 15.25	277.08 ± 19.93	317.88 ± 29.98	170.12 ± 9.55	192.23 ± 17.30	232.08 ± 11.51	-2.62 ± 9.22	84.85 ± 19.05	85.81 ± 35.23			
ACD (mm)			0.91 ± 0.01	0.96 ± 0.02	1.07 ± 0.03	0.92 ± 0.02	1.02 ± 0.02	1.10 ± 0.02	-0.01 ± 0.01	-0.07 ± 0.01	-0.02 ± 0.03			
CCT (µm)			175.85 ± 2.30	178.15 ± 2.14	181.10 ± 2.11	173.77 ± 2.02	174.73 ± 1.70	178.54 ± 1.82	2.08 ± 1.05	3.42 ± 1.42	2.56 ± 1.63			
Refraction (D)	2	LIH + HL	5.45 ± 0.15	6.72 ± 0.29	8.07 ± 0.44	5.57 ± 0.11	5.07 ± 0.08	4.72 ± 0.12	-0.11 ± 0.15	1.66 ± 0.28	3.35 ± 0.45	0.054	<0.001	0.066
		LIH + UnV	5.94 ± 0.16	7.07 ± 0.27	7.21 ± 0.24	5.72 ± 0.16	5.87 ± 0.24	4.94 ± 0.09	0.23 ± 0.17	1.20 ± 0.26	2.27 ± 0.26			
		LIH + HL + UnV	5.61 ± 0.14	5.87 ± 0.31	7.47 ± 0.36	5.48 ± 0.14	5.05 ± 0.18	5.05 ± 0.19	0.13 ± 0.14	0.83 ± 0.30	2.43 ± 0.37			
Axial length (mm)		LIH + HL	7.46 ± 0.03	7.29 ± 0.04	7.51 ± 0.06	7.46 ± 0.03	7.64 ± 0.03	7.97 ± 0.05	0.00 ± 0.02	-0.35 ± 0.03	-0.46 ± 0.03	0.557	<0.001	0.584
		LIH + UnV	7.42 ± 0.06	7.30 ± 0.06	7.48 ± 0.06	7.43 ± 0.06	7.63 ± 0.04	7.88 ± 0.05	-0.01 ± 0.01	-0.33 ± 0.04	-0.40 ± 0.04			
		LIH + HL + UnV	7.61 ± 0.07	7.39 ± 0.06	7.67 ± 0.07	7.60 ± 0.05	7.72 ± 0.05	8.06 ± 0.07	0.02 ± 0.03	-0.33 ± 0.03	-0.39 ± 0.05			
Choroidal thickness (µm)		LIH + HL	100.65 ± 12.61	304.00 ± 28.23	323.50 ± 33.60	98.46 ± 10.43	179.92 ± 17.16	209.38 ± 14.11	2.19 ± 10.04	124.08 ± 22.24	114.12 ± 29.26	0.089	<0.001	0.34
		LIH + UnV	91.77 ± 7.62	27.54 ± 16.73	284.85 ± 21.08	92.46 ± 8.06	208.23 ± 11.76	232.54 ± 7.10	-0.69 ± 5.77	62.31 ± 16.23	52.31 ± 23.14			
		LIH + HL + UnV	129.23 ± 17.86	223.05 ± 23.15	263.14 ± 19.82	126.27 ± 15.98	181.59 ± 17.76	197.36 ± 13.71	2.95 ± 11.64	41.45 ± 16.32	65.77 ± 10.06			

ACD (mm)		LIH + HL	0.93 ± 0.01	0.91 ± 0.02	1.03 ± 0.02	0.94 ± 0.01	1.01 ± 0.01	1.11 ± 0.02	-0.01 ± 0.02	-0.09 ± 0.02	-0.09 ± 0.02	0.08	<0.001	0.413
		LIH + UnV	0.93 ± 0.01	0.90 ± 0.01	1.03 ± 0.02	0.94 ± 0.01	1.00 ± 0.01	1.15 ± 0.02	-0.01 ± 0.01	-0.10 ± 0.01	-0.11 ± 0.03			
		LIH + HL + UnV	0.95 ± 0.01	0.92 ± 0.02	1.04 ± 0.02	0.96 ± 0.01	1.00 ± 0.02	1.12 ± 0.02	0.00 ± 0.01	-0.09 ± 0.02	-0.08 ± 0.01			
CCT (μm)		LIH + HL	162.69 ± 2.18	168.54 ± 1.56	173.69 ± 1.97	160.38 ± 2.13	166.23 ± 1.61	170.23 ± 2.25	2.31 ± 1.50	2.31 ± 0.87	3.46 ± 2.01	0.676	0.054	0.497
		LIH + UnV	165.88 ± 1.08	173.63 ± 1.31	175.75 ± 1.71	166.88 ± 0.93	169.58 ± 0.93	174.02 ± 1.63	-1.00 ± 0.78	4.06 ± 0.70	1.73 ± 1.39			
		LIH + HL + UnV	165.00 ± 1.96	172.80 ± 1.30	175.80 ± 2.27	164.14 ± 1.89	169.55 ± 1.53	174.68 ± 1.80	0.86 ± 1.30	3.25 ± 0.82	1.11 ± 1.55			
Refraction (D)	4	LIH + HL	5.34 ± 0.17	7.07 ± 0.15	9.34 ± 0.38	5.25 ± 0.16	5.34 ± 0.17	5.15 ± 0.16	0.08 ± 0.10	1.73 ± 0.26	4.19 ± 0.34	<0.001	<0.001	0.035
		LIH + UnV	5.34 ± 0.21	5.88 ± 0.37	7.20 ± 0.38	5.18 ± 0.22	4.90 ± 0.18	5.20 ± 0.15	0.16 ± 0.20	0.98 ± 0.39	2.00 ± 0.30			
		LIH + HL + UnV	5.22 ± 0.23	6.18 ± 0.26	8.24 ± 0.56	5.15 ± 0.20	5.25 ± 0.27	5.93 ± 0.14	0.07 ± 0.24	0.93 ± 0.44	2.30 ± 0.59			
Axial length (mm)		LIH + HL	7.43 ± 0.03	7.36 ± 0.03	7.55 ± 0.04	7.40 ± 0.03	7.64 ± 0.03	8.06 ± 0.04	0.03 ± 0.03	-0.28 ± 0.03	-0.51 ± 0.04	0.008	<0.001	<0.001
		LIH + UnV	7.61 ± 0.03	7.48 ± 0.05	7.72 ± 0.04	7.59 ± 0.04	7.72 ± 0.05	8.01 ± 0.04	0.02 ± 0.02	-0.24 ± 0.02	-0.29 ± 0.02			
		LIH + HL + UnV	7.64 ± 0.05	7.53 ± 0.06	7.80 ± 0.07	7.62 ± 0.06	7.75 ± 0.06	8.10 ± 0.05	0.02 ± 0.02	-0.22 ± 0.03	-0.31 ± 0.03			
Choroidal thickness (μm)		LIH + HL	108.31 ± 14.74	256.62 ± 21.03	333.42 ± 25.54	112.92 ± 15.37	161.42 ± 10.08	218.31 ± 15.38	-4.62 ± 8.23	95.19 ± 24.92	115.12 ± 26.59	0.109	<0.001	0.387
		LIH + UnV	115.38 ± 12.45	163.88 ± 16.66	238.62 ± 23.79	115.42 ± 11.93	128.46 ± 10.11	200.38 ± 14.37	-0.04 ± 8.13	35.42 ± 19.15	38.23 ± 23.45			
		LIH + HL + UnV	114.50 ± 12.53	267.08 ± 22.23	272.50 ± 22.83	113.79 ± 10.19	183.83 ± 15.53	210.83 ± 14.02	0.71 ± 5.67	83.25 ± 13.98	61.67 ± 23.04			
ACD (mm)		LIH + HL	0.91 ± 0.02	0.90 ± 0.02	1.06 ± 0.03	0.92 ± 0.02	1.01 ± 0.01	1.18 ± 0.04	-0.01 ± 0.01	-0.11 ± 0.02	-0.12 ± 0.06	0.115	<0.001	0.507
		LIH + UnV	0.92 ± 0.01	0.94 ± 0.01	1.05 ± 0.02	0.93 ± 0.01	1.02 ± 0.01	1.11 ± 0.02	-0.01 ± 0.01	-0.08 ± 0.01	-0.05 ± 0.02			
		LIH + HL + UnV	0.92 ± 0.01	0.91 ± 0.02	1.04 ± 0.02	0.93 ± 0.01	1.01 ± 0.04	1.15 ± 0.03	-0.01 ± 0.01	-0.10 ± 0.02	-0.11 ± 0.03			
CCT (μm)		LIH + HL	164.08 ± 0.98	169.54 ± 1.42	172.15 ± 1.82	164.54 ± 1.35	165.31 ± 1.67	170.27 ± 1.99	-0.46 ± 0.84	4.23 ± 0.84	1.88 ± 1.63	0.773	0.019	0.695
		LIH + UnV	169.27 ± 1.06	173.35 ± 1.39	177.40 ± 1.40	167.08 ± 1.07	170.48 ± 1.10	174.38 ± 1.64	2.19 ± 0.82	2.87 ± 0.79	3.01 ± 1.04			
		LIH + HL + UnV	163.54 ± 1.08	168.96 ± 1.03	172.71 ± 1.48	162.96 ± 0.91	165.79 ± 1.07	170.06 ± 1.49	0.58 ± 0.88	3.17 ± 0.71	2.65 ± 1.50			

		LIH + HL	4.35 ± 0.04	6.08 ± 0.44	10.16 ± 0.42	4.49 ± 0.09	3.84 ± 0.27	3.99 ± 0.20	-0.13 ± 0.07	2.24 ± 0.49	6.17 ± 0.47			
Refraction (D)		LIH + UnV	4.58 ± 0.12	5.28 ± 0.50	6.02 ± 0.43	4.47 ± 0.11	4.77 ± 0.24	4.72 ± 0.34	0.11 ± 0.13	0.50 ± 0.58	1.30 ± 0.42	<0.001	<0.001	<0.001
		LIH + HL + UnV	4.22 ± 0.15	5.53 ± 0.34	5.98 ± 0.50	4.58 ± 0.21	4.46 ± 0.20	4.00 ± 0.20	-0.36 ± 0.18	1.08 ± 0.43	1.98 ± 0.48			
		LIH + HL	7.52 ± 0.02	7.29 ± 0.02	7.33 ± 0.04	7.53 ± 0.02	7.62 ± 0.04	7.89 ± 0.02	-0.01 ± 0.02	-0.32 ± 0.03	-0.56 ± 0.03			
Axial length (mm)		LIH + UnV	7.58 ± 0.06	7.61 ± 0.05	7.94 ± 0.07	7.56 ± 0.06	7.73 ± 0.05	8.02 ± 0.07	0.02 ± 0.02	-0.11 ± 0.03	-0.08 ± 0.02	<0.001	<0.001	<0.001
		LIH + HL + UnV	7.47 ± 0.04	7.38 ± 0.03	7.73 ± 0.05	7.47 ± 0.03	7.55 ± 0.03	7.92 ± 0.05	0.00 ± 0.02	-0.17 ± 0.03	-0.19 ± 0.03			
		LIH + HL	107.54 ± 12.68	252.88 ± 19.26	329.19 ± 26.81	101.96 ± 15.18	154.77 ± 9.37	203.77 ± 11.29	5.58 ± 10.38	98.12 ± 19.50	125.42 ± 27.49			
Choroidal thickness (µm)	6	LIH + UnV	110.46 ± 19.74	183.25 ± 19.77	220.46 ± 23.98	106.96 ± 16.14	149.42 ± 12.87	191.08 ± 16.69	3.50 ± 6.38	33.83 ± 14.16	29.38 ± 19.45	0.012	<0.001	0.096
		LIH + HL + UnV	100.73 ± 12.13	180.69 ± 20.21	222.31 ± 16.41	93.38 ± 10.57	146.15 ± 10.68	179.62 ± 12.46	7.35 ± 9.08	34.54 ± 20.21	42.69 ± 14.79			
		LIH + HL	0.96 ± 0.01	0.90 ± 0.02	1.01 ± 0.03	0.96 ± 0.02	1.00 ± 0.02	1.12 ± 0.02	-0.01 ± 0.01	-0.10 ± 0.02	-0.10 ± 0.03			
ACD (mm)		LIH + UnV	0.93 ± 0.01	0.93 ± 0.01	1.04 ± 0.02	0.94 ± 0.01	1.01 ± 0.01	1.11 ± 0.02	-0.01 ± 0.01	-0.08 ± 0.01	-0.06 ± 0.02	0.14	<0.001	0.48
		LIH + HL + UnV	0.91 ± 0.01	0.93 ± 0.01	1.06 ± 0.03	0.92 ± 0.01	1.02 ± 0.01	1.14 ± 0.02	-0.01 ± 0.01	-0.09 ± 0.01	-0.07 ± 0.03			
		LIH + HL	167.69 ± 1.97	177.73 ± 2.04	179.35 ± 2.90	169.23 ± 1.85	173.85 ± 1.85	177.77 ± 2.25	-1.54 ± 1.25	3.88 ± 1.38	1.58 ± 1.82			
CCT (µm)		LIH + UnV	171.25 ± 1.46	178.19 ± 1.73	180.74 ± 2.16	171.00 ± 1.39	174.88 ± 1.01	178.17 ± 1.94	0.25 ± 0.57	3.31 ± 1.22	2.58 ± 1.10	0.654	0.001	0.762
		LIH + HL + UnV	169.96 ± 1.42	173.85 ± 1.54	176.63 ± 1.57	169.15 ± 1.45	170.02 ± 1.55	174.40 ± 1.67	0.81 ± 0.69	3.83 ± 0.75	2.22 ± 1.30			

All values for experimental and control eyes are expressed as the absolute value ± SEM; All P values represent statistical significance of interocular difference (experimental - control eye); IOD: intraocular difference, 2W RM ANOVA: Two way repeated measures analysis of variance, LIH: Lens induced hyperopia, HL: High intensity light, UnV: unrestricted vision, ACD: Anterior chamber depth, CCT: Central corneal thickness

Supplementary Table S2: Studies on lens-induced hyperopia, effects of light and unrestricted vision in animal models. Articles are listed by year of publication.

Author and year	Animal model	Animal age	Experimental protocol duration	Defocussing lens (Duration)	Background Light intensity	Light source	Intervention (Duration)	Outcome	Key findings	Dose response	Light and UnV interaction
Nathan et al 1984[1]	Cat	3 weeks	16 weeks	+6 D (8h/day)	NR	NR	-	No change in RE	Hyperopia cannot be induced reliably in kittens by defocus	No	No
Schaeffel et al 1988[2]	Chick	7 days	35 days	+2 and +4 D (14 h/day)	NR	NR	-	Change in RE: 2-3 D	Positive lenses wearing produce a consistent shift towards hyperopia	No	No
Schaeffel & Howland, 1991[3]	Chick	1 day	17 days	+4 D (12 h/day)	Irradiance: 0.3 W/m^2	Fluorescent tube + 60-Watt sodium lamp	-	Change in RE: 2.6-3.2 D, AL: 0.423 mm	Positive lenses produce strong shifts towards hyperopia	No	No
				+8 D (12 h/day)				Change in RE: 4.6 D, AL: 0.423 mm			
Irving et al 1992[4]	Chick	1 day	7 days	+30 D (14 h/day)	NR	Fluorescent lamp	-	IOD in RE: 17.7 D and AL: -0.04 mm	Refractive state of the developing chick eye is very plastic. During this developmental period, eyes can respond linearly to myopic defocus between +5 and +20 D, beyond which the relationship is non-linear.	No	No
				+20 D (14 h/day)				IOD in RE: 17.42 D and AL: -0.88 mm			
				+15 D (14 h/day)				IOD in RE: 13.91 D and AL: -0.48 mm			
				+10 D (14 h/day)				IOD in RE: 9.1 D and AL: -0.36 mm			
				+5 D (14 h/day)				IOD in RE: 4.75 D and AL: -0.08 mm			
Bartmann et al 1994[5]	Chick	5-10 days	4 days	+4 D (12 h/day)	1000-3000 lux	60-Watt bulb	1000-3000 lux (12 h/day)	RE: 7.97-8.9 D, AL: 8.54 mm	Constant light (24 h) does not alter lens induced hyperopia differently from 12 h of light.	Yes, for light duration	No
		12-16 days	4 days	+4 D (24 h/day)			1000-3000 lux (24 h/day)	RE: 10.7 D, AL: 8.61 mm			
Hung et al 1995[6]	Rhesus monkey	21-32 days	72-113 days	+3 D (12 h/day)	NR	NR	-	Increase in RE: 1.5 D	+3 D compensated more for the imposed defocus than +6 D	No	No
				+6 D (12 h/day)			-	Increase in RE: 1.25 D			
Wallman et al 1995[7]	Chick	4 days	4.5 days	+6 D (14 h/day)	NR	NR	-	Change in RE: 2.6 D	In eyes with myopic defocus, the choroid expands, pushing the retina forward, thereby partially correcting the imposed myopia. Lens removal produces rapid	No	No
				+15 D (14 h/day)				Change in RE: 7.2 D			

		8.5 days	4-11 days	-			UnV (14 h/day)	Almost emmetropic	thinning and reversal of hyperopia.		
Wildsoet & Wallman, 1995[8]	Chick	3 days	5 days	+6 D (14 h/day)	NR	Fluorescent lighting	-	IOD in RE: 6.7 D	Myopic defocus imposed by positive lenses resulted in increase in choroidal thickness, a slight decrease in ocular elongation, leading in hyperopia.	No	No
				+15 D (14 h/day)				IOD in RE: 16.3 D			
		8 days	4 days	-			UnV (14 h/day)	Reduction in RE: 3.8 D			
Diether & Schaeffel, 1997[9]	Chick	11 days	5 days	+6.9 D (12 h/day)	NR	60-Watt bulb	-	IOD in RE: 7.22 D and AL: -0.34 mm	Positive lenses cause large change in refraction towards hyperopia.	No	No
Guo et al 1996[10]	Chick	1 day	14 days	+10 D (24 h/day)	70-140 lux	Fluorescent lighting	70-140 lux (24 h/day)	IOD in RE: 5.0 D and AL: -0.27 D	Retinal defocus induced by plus lenses could induce experimental hyperopia in chicks kept under continuous light	No	No
Schmid & Wildsoet, 1996[11]	Chick	Young (1-10 days) & old (7-11 days)	5 days	+10 D (1 h/day)	250 lux	Fluorescent lamp	UnV (11 h/day)	IOD in RE: 2.3 D and AL: -0.13 mm	Hyperopia resulted from +10 D lenses, although the magnitude of hyperopia decreased as the duration of lens wear was decreased in a dose-dependent manner.	Yes, for UnV duration	No
				+10 D (3 h/day)			UnV (9 h/day)	IOD in RE: 4.8 D and AL: -0.15 mm			
				+10 D (6 h/day)			UnV (6 h/day)	IOD in RE: 6.0 D and AL: -0.21 mm			
				+10 D (9 h/day)			UnV (3 h/day)	IOD in RE: 7.4-7.6 D and AL: -0.24 to -0.37 mm			
				+10 D (12 h/day)			-	IOD in RE: 7.0-8.3 D and AL: -0.27 to -0.43 mm			
Schmid & Wildsoet, 1997[12]	Chick	1 day	9 days	+1 D (10 h/day)	250 lux	Fluorescent lights	-	Change in RE, day 6: -0.3 D and day 9: -2.1 D	+1 D focusing errors imposed is similar to the estimated depth of focus of the chick eye, which is below the threshold for compensatory responses	No	No
Nevin et al 1998[13]	Chick	1 week	4-5 days	+15 D (12 h/day)	900 lux	Fluorescent lighting	-	IOD in RE: 12.95-13.0, VCD: -0.45 mm and CT: 380 µm	Compensation to myopic defocus declines beyond +15 D, with +50 D lens having no apparent effect on eye growth and refraction and the +65 D lens inducing myopia instead of hyperopia.	No	No
				+30 D (12 h/day)				IOD in RE: 10.12 D and CT: 600 µm			
				+40 D (12 h/day)				IOD in RE: 10.38 D and VCD: -0.46 mm			
				+50 D (12 h/day)				IOD in RE: 3.88 D and CT: -50 µm			

				+65 D (12 h/day)				IOD in RE: -6.44 D and CT: -60 µm			
Graham & Judge, 1999[14]	Marmoset	4 weeks	4 weeks	+4 D (NR)	NR	NR	-	No hyperopia	High-plus lens produces slight relative hyperopia, whereas a low-plus lens has no effect on refraction.	No	No
				+8 D (NR)				RE: 0.99 D and AL: -0.09 mm			
Smith & Hung, 1999[15]	Rhesus monkey	2-4 weeks	10-23 weeks	+3, +6, +9, +12 D (12 h/day)	NR	NR	-	Compared to the control eyes, 3 D treated eyes were most hyperopic followed by 6, 9 and 12 D	Plus lens treated eyes maintained their initial degree of hyperopia or showed absolute hyperopic shifts. The hyperopia exhibited systematic reductions on lens removal.	No	No
		-	18-40 weeks	-			UnV (12 h/day)				
Priolo et al 2000[16]	Chick	1 day	7 days	+10 D (14 h/day)	NR	Fluorescent lighting	-	IOD in RE: 6.6 D	Myopic defocus produces relative hyperopia	No	No
Whatham & Judge, 2001[17]	Marmoset	56-94 days	35-64 days	+2 D (8 h/day)	NR	NR	-	IOD in RE: 2.5 D and VCD: -0.08 mm	Eyes develop hyperopia to positive soft contact lenses	No	No
				+4 D (8 h/day)				IOD in RE: 2.38 D and VCD: -0.12 mm			
Winawer and Wallman, 2002[18]	Chick	6-7 days	3 days	+7 D (14 h/day)	NR	Fluorescent lamp	-	IOD in RE: 6.9 D and AL: -0.156 mm	Visual feedback system regulating eye growth is exquisitely sensitive to brief periods of myopic defocus. Even minutes of daily lens wear can produce quite robust compensatory eye growth in hyperopic direction. The amount of compensational hyperopia is not predicted by the amount of positive lens-wear. Effect of periods of myopic defocus and resultant hyperopia is non-linear. Change in rates of ocular elongation and in choroid thickness, do not always occur in tandem.	Yes, for UnV duration	No
				+6 D (30 min/day, 7×)			UnV (10.5 h/day)	IOD in AL: -0.232 mm			
				+6 D (30 min/day, 2×)			UnV (13 h/day)	IOD in AL: -0.181 mm			
				+6 D (2 min/day, 14×)			UnV (13.5 h/day)	IOD in RE: 3.4 D and AL: -0.118 mm			
				+6 D (7 min/day, 4×)			UnV (13.5 h/day)	IOD in AL: -0.169 mm			
			4 days	+6.7 D (2 min/day, 14×)			UnV (13.5 h/day)	IOD in RE: 3.9 D and AL: -0.219 mm			
				+6.7 D (28 min/day, 1×)			UnV (13.5 h/day)	IOD in RE: 3.7 D and AL: -0.166 mm			
			3 days	+6 D (20 secs/day, 42×)			UnV (13.8 h/day)	IOD in RE: 0.1 D and AL: -0.065 mm			
				+6 D (5 secs/day, 168×)			UnV (13.8 h/day)	IOD in RE: 1.0 D and AL: -0.041 mm			
				+6 D (2 secs/day, 420×)			UnV (13.8 h/day)	IOD in RE: -0.1 D and AL: 0.01 mm			
Park et al 2003[19]	Chick	6-7 days	3 days	+2.7-3 D (14 h/day)	NR	Fluorescent lighting	Restrained in drum (fixed distance) or	IOD in RE: 3.7 D, AL: -0.79 mm and CT: 86 µm	Compensation for imposed myopic defocus does not depend simply on the amount of defocus	No	No

				+6.7-10 D (14 h/day)<br>+14.7-18 D (14 h/day)			free in cages (variable distance)	IOD in RE: 4.1-5.1 D, AL: -0.116 mm and CT: 111 µm<br>IOD in RE: 1.0-7.0 D, AL: 0.010 to -0.168 mm and CT: 50-100 µm	(quantity of blur) or by the duration of sharp vision. Eye can discern the blur and use it to guide eye growth during lens compensation.		
Smith et al 2003[20]	Rhesus monkey	3 weeks	7 months	+3 D (12 h/day)<br>+3 D (24 h/day)	230-630 lux	NR	230-630 lux (12 h/day)<br>230-630 lux (24 h/day)	Change in RE: 2.3 D<br>Change in RE: 1.6 D	Eyes treated with +3 D lenses were more hyperopic (difference from control in RE: 2.2 D), irrespective of ambient lighting cycle	Yes, for light duration	No
Zhu et al 2003[21]	Chick	1 week	3 days	+6 D (30 min/day, 2×)<br>+6 D (15 min/day, 4×)<br>+6 D (10 min/day, 6×)<br>+6 D (2 min/day, 6×)<br>+10 D (2 min/day, 6×)	NR	Fluorescent lamp	UnV (11 h /day)<br>UnV (11 h /day)<br>UnV (11 h /day)<br>UnV (11.8 h /day)<br>UnV (11.8 h /day)	IOD in RE: 1.1-2.0 D and AL: -0.011 to -0.067 mm<br>IOD in RE: 3.5 D and AL: -0.076 mm<br>IOD in RE: 2.9 D and AL: -0.085 mm<br>IOD in RE: 1.4 D and AL: -0.069 mm<br>IOD in RE: 2.1 D and AL: -0.054 mm	Brief periods of myopic defocus imposed by positive lenses with UnV the remainder of the time caused eyes to become hyperopic and reduced the rate of ocular elongation.	Yes, for UnV duration	No
Nickla et al 2005[22]	Chick	12 days	5 days	+10 D (2 h/day)<br>+10 D (3 h/day)<br>+10 D (6 h/day)	NR	NR	UnV (10 h/day)<br>UnV (9 h/day)<br>UnV (6 h/day)	IOD in RE: 3.9 D, AL: -0.07 mm, CT: -39 µm<br>IOD in AL: -0.088 mm and CT: 77 µm<br>IOD in RE: 3.9 D, AL: -0.113 mm and CT: 183 µm	Positive lens wear resulted in a rapid increase in hyperopia, a decrease in AL and an increase in CT, the magnitude of which was a linear function of the duration of exposure.	Yes, for UnV duration	No
Winawer et al 2005[23]	Chick	6-7 days	3 days	+2.7 D myopic defocus (4 h/day)	1400 lux	Red LEDs	-	IOD in RE: 2.37 D and AL: -0.003 mm	Low myopic defocus induces low hyperopia, but no changes in ocular elongation	No	No
Zhu et al 2005[24]	Chick	6-10 days	2 hours-2 days	+10 D (10 min)<br>+10 D (1 h)	NR	NR	-	Change in AL: 0.008 mm and CT: 23 µm<br>Change in AL: 0.048 mm and CT: 73 µm	Eyes require only a brief period of positive lens wear to initiate compensation	No	No
Nickla 2007[25]	Chick	5-7 days	4 days	+10 D	Darkness	NR	300 lux (0.5 h/day)	Difference from control in RE: 3.6 D, AL: -0.419 mm, CT: 35 µm	Compensatory responses to myopic defocus are much more robust under normal light/dark cycle than in darkness. Frequent	No	No

				+10 D	Darkness		300 lux (2 min/hour for 14 h)	IOD in RE: 5.2 D and AL: -0.178 mm	brief episodes of myopic defocus under darkness show ocular growth inhibition without choroidal thickening		
				+10 D	Darkness		300 lux (1 min/2 hour for 14 h)	IOD in RE: -0.3 D and AL: -0.008 mm			
Padmanabhan et al 2007[26]	Chick	11-13 days	7 days	+10 D (12 h/day)	331-385 lux	NR	331-385 lux (12 h/day)	IOD in RE: 8.25 D and AL: -0.379 mm	Constant light impairs emmetropization and accelerates lens compensation. Both light groups recovered from LIH on lens removal.	Yes, for light and UnV duration	No
				+10 D (24 h/day)			331-385 lux (24 h/day)	IOD in RE: 10.5 D and AL: -0.493 mm			
		14-21 days	7 days	-			UnV + 331-385 lux (12 h/day)	IOD in RE: -8.25 D and AL: 0.575 mm			
				-			UnV + 331-385 lux (24 h/day)	IOD in RE: -9.0 D and AL: 0.537 mm			
Shen & Sivak, 2007[27]	Tilapia	NR	2 weeks	+15 D (12 h/day)	NR	Fluorescent lighting	-	IOD in RE: 6.25-8.05 D and AL: -0.12 mm	Fishes exhibit positive lens-induced hyperopia and recovery on lens removal	No	No
			2 weeks	-			UnV (12 h/day)	IOD in RE ~0.0 D			
Kee & Deng, 2008[28]	Chick	5 days	7 days	+10 D (12 h/day)	300 lux	NR	-	IOD in RE: 9.88-11.48 D	Myopic defocus produces relative hyperopia	No	No
Metlapally & McBrien, 2008[29]	Tree Shrew	14 days of VE	6-8 weeks	+4 D (14 h/day)	265 lux	NR	-	Difference from control in RE: 6.9 D	+4 D lens produces relative hyperopia but not +6 or +9.5 D lens wear.	No	No
			6-8 weeks	+6 D (14 h/day)				Difference from control in RE: ~ 0 D			
			6-8 weeks	+9.5 D (14 h/day)							
Howlett & McFadden, 2009[30]	Guinea pig	2-3 days	10 days	+2 D (12 h/day)	NR	Fluorescent lighting	-	IOD in RE: -1.1 D and AL: -0.04mm	Short periods of lens-wear were sufficient to alter the ocular development of the guinea pig in a manner that was dependent on the magnitude of the lens worn.	No	No
				+4 D (12 h/day)				IOD in RE: -0.5 D and AL: -0.03 mm			
Troilo et al, 2009[31]	Marmoset	41-101 days	10 weeks	+3 D CL (NR; removed overnight)	NR	NR	-	IOD in RE: 1.40 D and VCD: -0.082 mm	Positive power lenses produced a significant reduction in VCD and hyperopia relative to the contralateral control eyes	No	No
Zhu and Wallman, 2009[32]	Chick	1 week	3 days	+7 D (14 h/day)	NR	Fluorescent lighting	Constant myopic defocus under light	Relative change in RE: 6.3 D, AL: -0.195 mm, CT: -99 μm	Effects of episodes of defocus rise rapidly with episode duration to an asymptote and decline between episodes.	Yes, for light duration	No

				+7 D (10 sec/10 min)			Periods of myopic defocus under light, darkness otherwise	Relative change in RE: -0.3 D, AL: -0.016 mm, CT: 10 µm	Emmetropization signals increase to saturation during episodes of defocus and then decline between episodes. The signal regulating the rate of ocular AL elongation and CT rises by 50% in 1–4 minutes of lens wear and falls by 50% in 24.4 hours.		
				+7 D (30 sec/1 h)				Relative change in RE: 0.7 D, m, AL: -0.077 mm, CT: -2 µm			
				+7 D (1 min/1 h)				Relative change in RE: 0.9 D mm, AL: -0.068 mm, CT: 24 µm			
				+7 D (2 min/1 h)				Relative change in RE: 0.7 D, mm, AL: -0.182 mm, CT: 26 µm			
				+7 D (2 min/10 min)				Relative change in RE: 3.6 D, AL: -0.211 mm, CT: 38 µm			
				+7 D (5 min/1 h)				Relative change in RE: 4.4 D, AL: -0.198 mm, CT: 57 µm			
				+7 D (10 min/1 h)				Relative change in RE: 5.4 D, AL: -0.153 mm, CT: 132 µm			
				+7 D (30 min/2 h)				Relative change in RE: NR, AL: -0.232 mm, CT: 71 µm			
				+7 D (30 min/4 h)				Relative change in RE: 6.6 D, AL: -0.210 mm, CT: 106 µm			
				+7 D (30 min/6 h)				Relative change in RE: 4.5 D, AL: -0.176 mm, CT: 84 µm			
				+7 D (30 min/12 h)				Relative change in RE: 2.9 D, AL: -0.204 mm, CT: -4 µm			
				+7 D (30 min/24 h)				Relative change in RE: 2.2 D, AL: -0.139 mm, CT: 19 µm			
				+7 D (30 min/48 h)				Relative change in RE: 2.4 D, AL: -0.063 mm, CT: 29 µm			
Ashby & Schaeffel, 2010[33]	Chick	7 days	5 days	+7 D (12 h/day)	500 lux	Fluorescent + Quartz-	15,000 lux (5 h/day)	IOD in RE: 5.4 D and AL: -0.19 mm	High-intensity light (15,000 lux) accelerated compensation for	No	No

						halogen lamp	-	IOD in RE: 5.4 D and AL: -0.13 mm	positive lenses, however end point refractions were similar		
Siegwart & Norton, 2010[34]	Tree Shrew	Young (11 days of VE)	34 days	+4 D (14 h/day)	NR	NR	-	RE: 3.8 D	Juvenile animals with emmetropia compensates poorly to myopic defocus compared to young animals with hyperopic refraction. On lens removal, eyes recovery rapidly with decrease toward emmetropia.	No	No
		Juvenile (24 days of VE)	12 days	+3 D (14 h/day)			-	Difference from control in RE: 1.3 D and AL: -0.031 mm			
			12 days	+5 D (14 h/day)			-	Difference from control in RE: 1.8 D and AL: -0.041 mm			
		45 days of VE	25 days	-			UnV (14 h/day)	RE: 0.0 D			
Tepelus & Schaeffel, 2010[35]	Chick	5 days	5 days	+7 D (12 h/day)	NR	NR	-	Change in RE: 6.05 D	Positive lenses induce hyperopia, which matches the power of the lenses and returned to their initial refractive states on lens removal	No	No
		10 days	5 days	-			UnV (12 h/day)	Change in RE: 6.1 D			
Benavente-Perez et al 2012[36]	Marmo set	70-76 days	12 weeks	+5 D (9 h/day)	700 lux	NR	-	RE: +5.65 D	Imposed myopic defocus induces hyperopia	No	No
Hammond & Wildsoet, 2012[37]	Chick	3 days	7 days	+10 D (12 h/day)	20 lux	LEDs	-	IOD in AL: -0.298 mm and CT: 25 µm	Compensation to imposed myopic defocus was superior under the brightest (200 lux) illuminance for the larger (+20 D) focusing error	Yes, for light intensity	No
					200 lux			IOD in AL: -0.194 mm and CT: -352 µm			
				+20 D (12 h/day)	20 lux			IOD in AL: 0.315 mm and CT: -120 µm			
					200 lux			IOD in AL: -0.194 mm and CT: 352 µm			
Tepelus et al 2012[38]	Chick	1 week	5 days	+7 D (12 h/day)	500 lux	NR	-	IOD in RE: 5.56 D and mean change in RE: 6.04 D	Myopic defocus induces hyperopic shift	No	No
Hammond et al 2013[39]	Chick	1 day	4 days	+5 D (12 h/day)	NR	NR	-	Change in RE: 4.46 D, AL: ~0.3 mm, CT: ~50 µm	Eye growth is not guided by the magnitude of the defocus. Overshoot seen with +6 D indicate a bang-bang controller	No	No
				+10 D, +15 D (12 h/day)				Change in RE: ~7-8 D, AL: ~0.2 mm, CT: ~300 µm			
			24 hours	Plano, +1 D, +2D, +3 D (24 h)				Change in CT: 7, 18, 80, 65 µm			
			2 days	+6 D (14 h/day)				Change in RE: 8.4 D			

Siegwart & Norton, 2013[40]	Tree Shrew	11 days of VE	13 days	+4 D (14 h/day)	500-1000 lux	NR	-	RE: 4.5 D	Normal rapid decrease in refractive state towards myopia, then eyes gradually compensated for plus lens	No	No
		24 days of VE	21 days	+4 D (2 h/day)			UnV (12 h/day)	RE: 2.7 D	Slightly hyperopic		
		11 days of VE	34 days	+4 D (14h/day)			-	RE: 3.8 D	Initial shifting of refraction to near-emmetropia, then towards myopia, and finally hyperopic		
Smith et al 2013[41]	Rhesus monkey	2-3 weeks	118 days	+3 D (12 h/day)	350 lux	NR	-	IOD in RE: +1.69 D and VCD: -0.33 mm	Myopic defocus develops compensating hyperopia	No	No
Zhu et al 2013[42]	Chick	1 week	3 days	+6, +7, +10 D (14 h/day)	NR	Fluorescent lighting	-	IOD in AL: -0.131 mm	Young, rapidly growing animal eyes can shorten axially to facilitate compensation for imposed myopic defocus	No	No
	Marmoset	4 months	14 days	+5 D CL (10 h/day)				8% of the eyes shortened axially			
				+3 D (10 h/day)				4% of the eyes shortened axially			
				+5 D (10 h/day)							
Jiang et al 2018[43]	Mice	P21	3 weeks	+5 D (12 h/day)	50 lux	Fluorescent lighting	-	IOD in RE: ~2.9 D and CT: ~0.01 mm	Low myopic defocus induces low hyperopia not significantly different from control eyes	No	No
Zheng et al 2018[44]	Chick	5 days	6-8 days	+10/-10 D (33:67) multizone dual power (12 h/day)	500 lux	Fluorescent + metal halide lamp	-	IOD in RE: -7.3 D and AL: 0.46 mm	Myopic defocus and bright light are additive against experimental myopia.	Yes, for light intensity	No
							10,000 lux (6 h/day)	IOD in RE: -2.75 D and AL: 0.11 mm			
							20,000 lux (6 h/day)	IOD in RE: 1.7 D and AL: -0.06 mm			
							40,000 lux (6 h/day)	IOD in RE: 1.7 D and AL: -0.14 mm			
Zhu et al 2022[45]	Marmoset	10 weeks	4 weeks	+5 D (9 h/day)	700 lux	Fluorescent lamp	-	IOD in VCD: -0.025 mm and RE: 1.24 D	Wearing +5 D continuously slowed eye growth, whereas interrupted defocus with UnV enhanced defocus compensation resulting in greater hyperopia	No	No
							UnV (0.5 h/day, 2×)	IOD in VCD: -0.086 mm and RE: 1.93 D			
Our Study, 2024	Chick	1 day	8 days	+10 D (12 h/day)	150 lux	LEDs	-	RE: 3.48 D, AL: -0.42 mm, CT: 85.81 µm	2, 4, or 6 hours of UnV slows LIH by promoting emmetropization in a duration-dependent manner. Light potentiates the drive for	Yes, for UnV and light duration	Yes
				+10 D (10 h/day)			UnV (2 h/day)	IOD in RE: 2.27 D, AL: -0.40 mm, CT: 52.31 µm			

				+10 D (8 h/day)			UnV (4 h/day)	IOD in RE: 2.0 D, AL: -0.29 mm, CT: 38.23 µm	hyperopia in a dose-dependent manner.		
				+10 D (6 h/day)			UnV (6 h/day)	IOD in RE: 1.3 D, AL: -0.08 mm, CT: 29.38 µm			
				+10 D (12 h/day)			15,000 lux (2 h/day)	IOD in RE: 3.35 D, AL: -0.46 mm, CT: 114.12 µm			
				+10 D (12 h/day)			15,000 lux (4 h/day)	IOD in RE: 4.19 D, AL: -0.51 mm, CT: 115.12 µm			
				+10 D (12 h/day)			15,000 lux (6 h/day)	IOD in RE: 6.17 D, AL: -0.56 mm, CT: 125.42 µm			
				+10 D (10 h/day)			UnV + 15,000 lux (2 h/day)	IOD in RE: 2.43 D, AL: -0.39 mm, CT: 65.77 µm			
				+10 D (8 h/day)			UnV + 15,000 lux (4 h/day)	IOD in RE: 2.3 D, AL: -0.31 mm, CT: 61.67 µm			
				+10 D (6 h/day)			UnV + 15,000 lux (6 h/day)	IOD in RE: 1.98 D, AL: -0.19 mm, CT: 42.69 µm			

UnV: Unrestricted vision, LIH: Lens induced hyperopia, IOD: Interocular difference, LED: Light emitting diode, RE: Refractive error, AL: Axial length, CT: Choroidal thickness, NR: Not recorded, CL: Contact lens, min: minute, h: hour, sec: second.
Relative Change = Change in experimental eye - Change in control eye

Supplementary figures

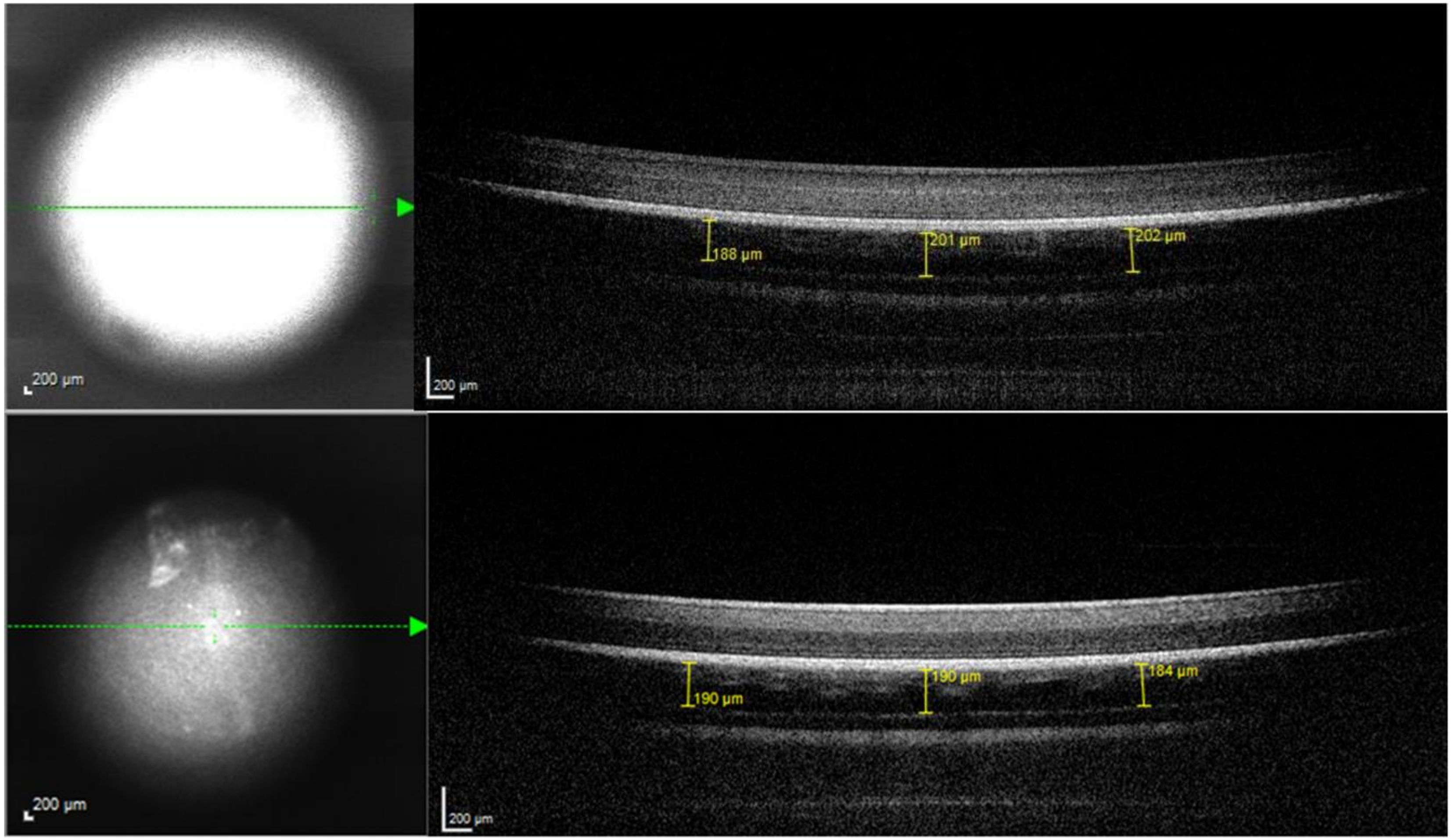

Supplementary Figure S1. Representative examples of posterior segment OCT scans and measurements of the choroidal thickness in chicken eyes on D8.

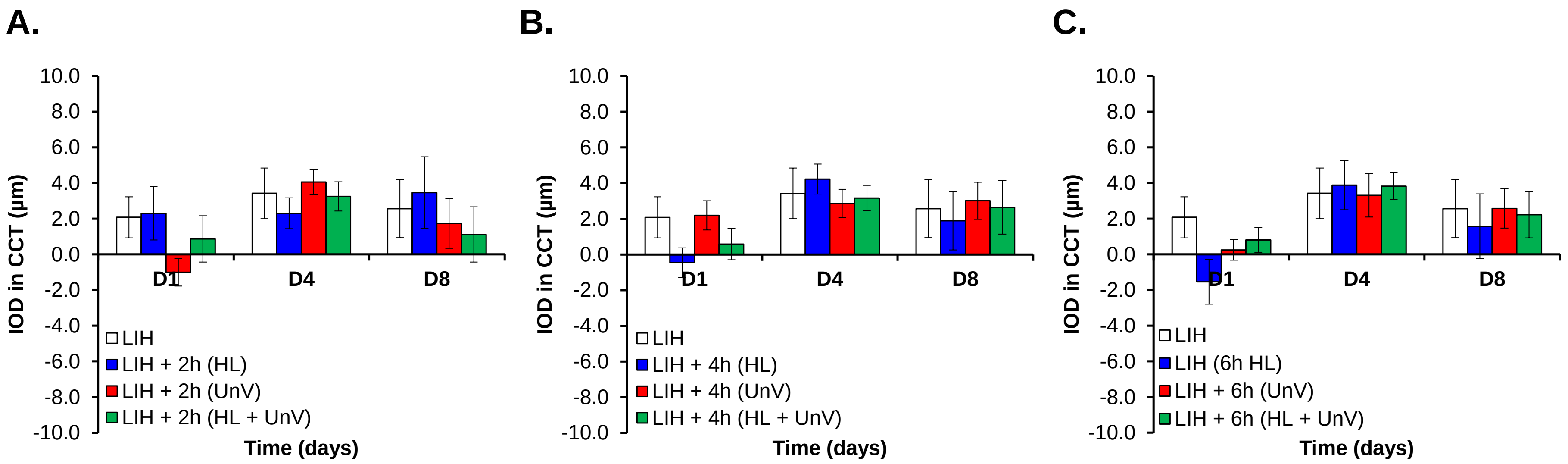

Supplementary Figure S2. IOD in CCT on days 1, 4, and 8 of the experimental protocol in the group not exposed to any intervention (LIH) and groups exposed to 2 hours (**A**), 4 hours (**B**), and 6 hours (**C**) of HL, UnV, or both (HL + UnV).

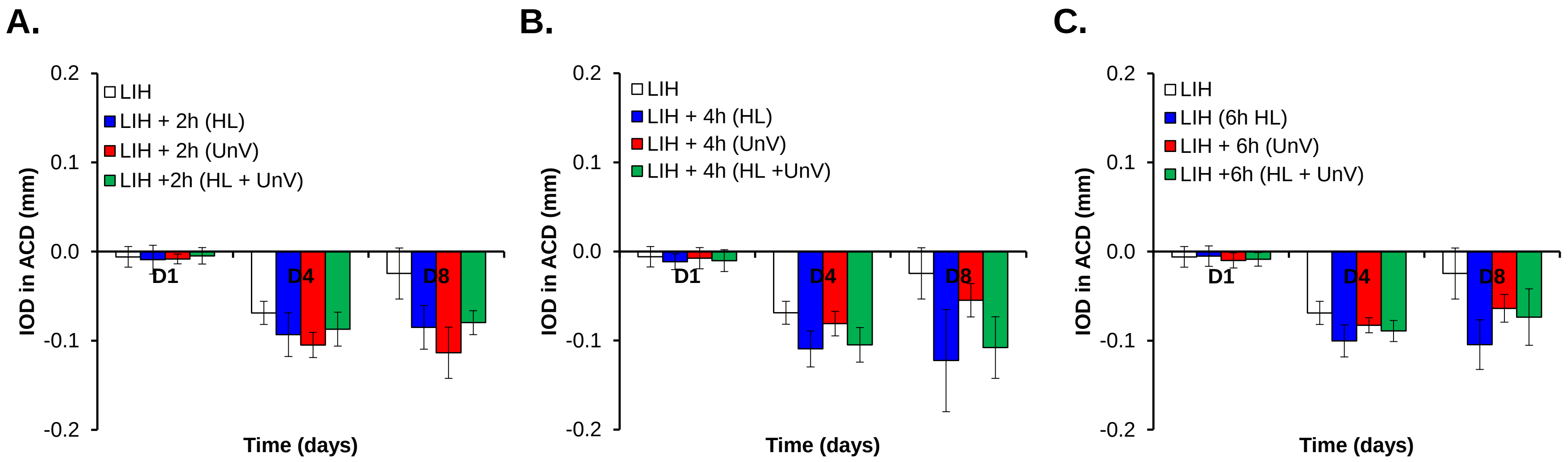

Supplementary Figure S3. IOD in ACD on days 1, 4, and 8 of the experimental protocol in the group not exposed to any intervention (LIH) and groups exposed to 2 hours (**A**), 4 hours (**B**), and 6 hours (**C**) of HL, UnV, or both (HL + UnV).

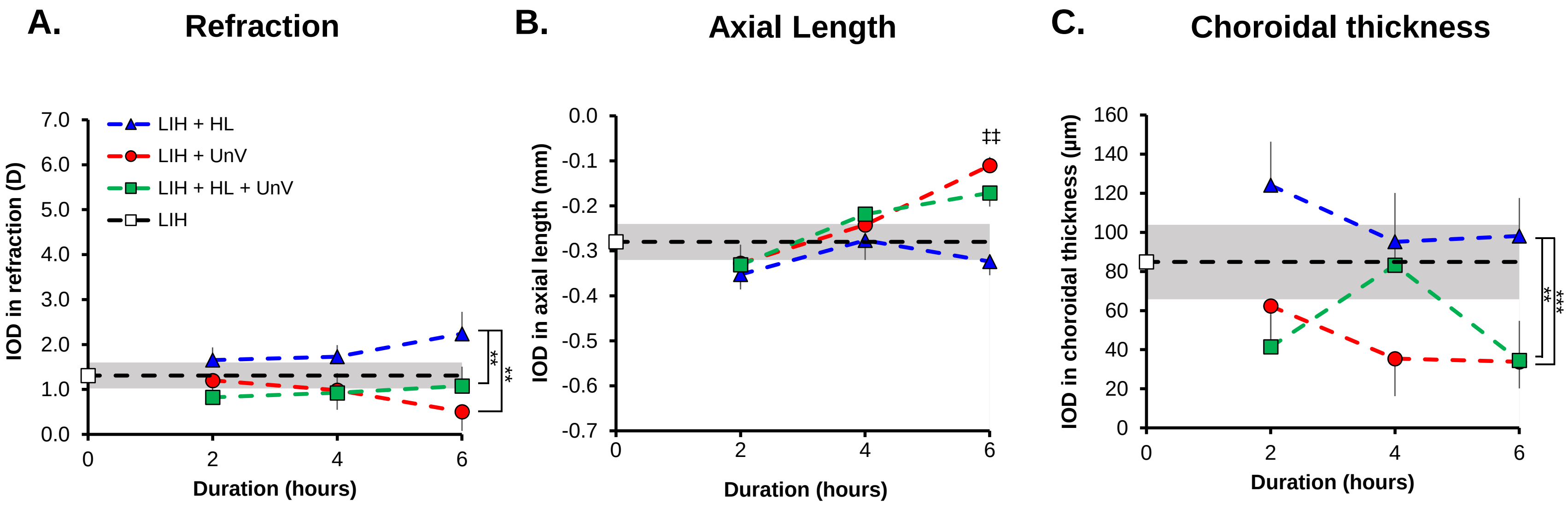

Supplementary Figure S4. Duration-response curve for the IOD in refraction (**A**), axial length (**B**), and choroidal thickness (**C**) in the groups exposed to 2, 4, and 6 hours of HL, UnV, or both (HL + UnV) on day 4 of the experimental protocol. The LIH group that was not exposed to any intervention is represented by a *white square* and a *shaded area* for mean ± 95% confidence interval. HL group is different from both UnV and HL + UnV groups at 6 hours: ‡‡P < 0.01. HL group is significantly different from both UnV and HL + UnV groups: **P* < 0.05, ***P* < 0.01, ***P < 0.001.

References

1. Nathan J, Crewther SG, Crewther DP, Kiely PM. Effects of retinal image degradation on ocular growth in cats. *Invest Ophthalmol Vis Sci* 1984;25:1300-1306.
2. Schaeffel F, Glasser A, Howland HC. Accommodation, refractive error and eye growth in chickens. *Vision Res* 1988;28:639-657.
3. Schaeffel F, Howland HC. Properties of the feedback loops controlling eye growth and refractive state in the chicken. *Vision Res* 1991;31:717-734.
4. Irving EL, Sivak JG, Callender MG. Refractive plasticity of the developing chick eye. *Ophthalmic Physiol Opt* 1992;12:448-456.
5. Bartmann M, Schaeffel F, Hagel G, Zrenner E. Constant light affects retinal dopamine levels and blocks deprivation myopia but not lens-induced refractive errors in chickens. *Vis Neurosci* 1994;11:199-208.
6. Hung LF, Crawford ML, Smith EL. Spectacle lenses alter eye growth and the refractive status of young monkeys. *Nat Med* 1995;1:761-765.
7. Wallman J, Wildsoet C, Xu A, et al. Moving the retina: choroidal modulation of refractive state. *Vision Res* 1995;35:37-50.
8. Wildsoet C, Wallman J. Choroidal and scleral mechanisms of compensation for spectacle lenses in chicks. *Vision Res* 1995;35:1175-1194.
9. Diether S, Schaeffel F. Local changes in eye growth induced by imposed local refractive error despite active accommodation. *Vision Res* 1997;37:659-668.
10. Guo SS, Sivak JG, Callender MG, Herbert KL. Effects of continuous light on experimental refractive errors in chicks. *Ophthalmic Physiol Opt* 1996;16:486-490.
11. Schmid KL, Wildsoet CF. Effects on the compensatory responses to positive and negative lenses of intermittent lens wear and ciliary nerve section in chicks. *Vision Res* 1996;36:1023-1036.
12. Schmid KL, Wildsoet CF. The sensitivity of the chick eye to refractive defocus. *Ophthalmic Physiol Opt* 1997;17:61-67.
13. Nevin ST, Schmid KL, Wildsoet CF. Sharp vision: a prerequisite for compensation to myopic defocus in the chick? *Curr Eye Res* 1998;17:322-331.
14. Graham B, Judge SJ. The effects of spectacle wear in infancy on eye growth and refractive error in the marmoset (Callithrix jacchus). *Vision Res* 1999;39:189-206.
15. Smith EL, 3rd, Hung LF. The role of optical defocus in regulating refractive development in infant monkeys. *Vision Res* 1999;39:1415-1435.
16. Priolo S, Sivak JG, Kuszak JR, Irving EL. Effects of experimentally induced ametropia on the morphology and optical quality of the avian crystalline lens. *Invest Ophthalmol Vis Sci* 2000;41:3516-3522.
17. Whatham AR, Judge SJ. Compensatory changes in eye growth and refraction induced by daily wear of soft contact lenses in young marmosets. *Vision Res* 2001;41:267-273.
18. Winawer J, Wallman J. Temporal constraints on lens compensation in chicks. *Vision Res* 2002;42:2651-2668.
19. Park TW, Winawer J, Wallman J. Further evidence that chick eyes use the sign of blur in spectacle lens compensation. *Vision Res* 2003;43:1519-1531.
20. Smith EL, 3rd, Hung LF, Kee CS, Qiao-Grider Y, Ramamirtham R. Continuous ambient lighting and lens compensation in infant monkeys. *Optom Vis Sci* 2003;80:374-382.

21. Zhu X, Winawer JA, Wallman J. Potency of myopic defocus in spectacle lens compensation. *Invest Ophthalmol Vis Sci* 2003;44:2818-2827.
22. Nickla DL, Sharda V, Troilo D. Temporal integration characteristics of the axial and choroidal responses to myopic defocus induced by prior form deprivation versus positive spectacle lens wear in chickens. *Optom Vis Sci* 2005;82:318-327.
23. Winawer J, Zhu X, Choi J, Wallman J. Ocular compensation for alternating myopic and hyperopic defocus. *Vision Res* 2005;45:1667-1677.
24. Zhu X, Park TW, Winawer J, Wallman J. In a matter of minutes, the eye can know which way to grow. *Invest Ophthalmol Vis Sci* 2005;46:2238-2241.
25. Nickla DL. Transient increases in choroidal thickness are consistently associated with brief daily visual stimuli that inhibit ocular growth in chicks. *Exp Eye Res* 2007;84:951-959.
26. Padmanabhan V, Shih J, Wildsoet CF. Constant light rearing disrupts compensation to imposed- but not induced-hyperopia and facilitates compensation to imposed myopia in chicks. *Vision Res* 2007;47:1855-1868.
27. Shen W, Sivak JG. Eyes of a lower vertebrate are susceptible to the visual environment. *Invest Ophthalmol Vis Sci* 2007;48:4829-4837.
28. Kee CS, Deng L. Astigmatism associated with experimentally induced myopia or hyperopia in chickens. *Invest Ophthalmol Vis Sci* 2008;49:858-867.
29. Metlapally S, McBrien NA. The effect of positive lens defocus on ocular growth and emmetropization in the tree shrew. *J Vis* 2008;8:1.1-12.
30. Howlett MH, McFadden SA. Spectacle lens compensation in the pigmented guinea pig. *Vision Res* 2009;49:219-227.
31. Troilo D, Totonelly K, Harb E. Imposed anisometropia, accommodation, and regulation of refractive state. *Optom Vis Sci* 2009;86:E31-39.
32. Zhu X, Wallman J. Temporal properties of compensation for positive and negative spectacle lenses in chicks. *Invest Ophthalmol Vis Sci* 2009;50:37-46.
33. Ashby RS, Schaeffel F. The effect of bright light on lens compensation in chicks. *Invest Ophthalmol Vis Sci* 2010;51:5247-5253.
34. Siegwart JT, Jr., Norton TT. Binocular lens treatment in tree shrews: Effect of age and comparison of plus lens wear with recovery from minus lens-induced myopia. *Exp Eye Res* 2010;91:660-669.
35. Tepelus TC, Schaeffel F. Individual set-point and gain of emmetropization in chickens. *Vision Res* 2010;50:57-64.
36. Benavente-Perez A, Nour A, Troilo D. The effect of simultaneous negative and positive defocus on eye growth and development of refractive state in marmosets. *Invest Ophthalmol Vis Sci* 2012;53:6479-6487.
37. Hammond DS, Wildsoet CF. Compensation to positive as well as negative lenses can occur in chicks reared in bright UV lighting. *Vision Res* 2012;67:44-50.
38. Tepelus TC, Vazquez D, Seidemann A, Uttenweiler D, Schaeffel F. Effects of lenses with different power profiles on eye shape in chickens. *Vision Res* 2012;54:12-19.
39. Hammond DS, Wallman J, Wildsoet CF. Dynamics of active emmetropisation in young chicks--influence of sign and magnitude of imposed defocus. *Ophthalmic Physiol Opt* 2013;33:215-226.
40. Siegwart JTJ, Norton TT. Response to Interrupted Hyperopia After Restraint of Axial Elongation in Tree Shrews. 2013;90:131-139.
41. Smith EL, 3rd, Hung LF, Huang J, Arumugam B. Effects of local myopic defocus on refractive development in monkeys. *Optom Vis Sci* 2013;90:1176-1186.
42. Zhu X, McBrien NA, Smith EL, 3rd, Troilo D, Wallman J. Eyes in various species can shorten to compensate for myopic defocus. *Invest Ophthalmol Vis Sci* 2013;54:2634-2644.

43. Jiang X, Kurihara T, Kunimi H, et al. A highly efficient murine model of experimental myopia. *Sci Rep* 2018;8:2026.
44. Zheng H, Tse DY, Tang X, To C, Lam TC. The Interactions Between Bright Light and Competing Defocus During Emmetropization in Chicks. *Invest Ophthalmol Vis Sci* 2018;59:2932-2943.
45. Zhu X, Kang P, Troilo D, Benavente-Perez A. Temporal properties of positive and negative defocus on emmetropization. *Sci Rep* 2022;12:3582.
